# mTOR signaling pathway regulation HIF-1 α effects on LPS induced intestinal mucosal epithelial model damage

**DOI:** 10.1186/s12860-024-00509-5

**Published:** 2024-04-23

**Authors:** Zeyong Huang, Wenbin Teng, Liuxu Yao, Kai Xie, Suqin Hang, Rui He, Yuhong Li

**Affiliations:** 1 Department of Anesthesiology, Shulan (Hangzhou) Hospital, Shulan International Medical College, Zhejiang Shuren College, 310015 Hangzhou, China; 2 Department of Anesthesiology, the First Affiliated Hospital, College of Medicine, Zhejiang University, 310001 Hangzhou, China; 3 Rehabilitation Medicine Center, Department of Anesthesiology, Zhejiang Provincial People’s Hospital, Affiliated People’s Hospital, Hangzhou Medical College, 310014 Hangzhou, China; 4 Department of Anesthesiology, Shaoxing People’s Hospital, Zhejiang University, 312000 Shaoxing, China; 5 Department of Anesthesiology, Shulan (Hangzhou) Hospital, Shulan International Medical College, Shuren University, 848 Dongxin Road, Xiacheng District, 310004 Hangzhou, Zhejiang China

**Keywords:** Caco-2 cells, LPS induced intestinal epithelial model injury, Hypoxia-inducible factor-1α, mTOR/P70S6K signalling pathway, In vitro

## Abstract

**Background:**

Sepsis-induced small-intestinal injury is associated with increased morbidity and mortality. Our previous study and other papers have shown that HIF-1α has a protective effect on intestinal mucosal injury in septic rats. The purpose of this study is to further verify the protective effect of HIF-1α on intestinal mucosa and its molecular mechanism in vitro experiments.

**Methods:**

Caco-2 cells were selected and experiment was divided into 2 parts. Part I: HIF-1α activator and inhibitor were used to treat lipopolysacchrides (LPS)-stimulated Caco-2 cells respectively, to explore the effect of HIF-1α on LPS induced Caco-2 cell epithelial model; Part II: mTOR activator or inhibitor combined with or without HIF-1α activator, inhibitor to treat LPS-stimulated Caco-2 cells respectively, and then the molecular mechanism of HIF-1α reducing LPS induced Caco-2 cell epithelial model damage was detected.

**Results:**

The results showed that HIF-1α activator decreased the permeability and up regulated tight junction (TJ) expression, while HIF-1α inhibitor had the opposite effect with the HIF-1α activator. mTOR activation increased, while mTOR inhibition decreased HIF-1α protein and expression of its downstream target molecules, which can be attenuated by HIF-1α activator or inhibitor.

**Conclusion:**

This study once again confirmed that HIF-1α alleviates LPS-induced mucosal epithelial model damage through P70S6K signalling pathway. It is of great value to explore whether HIF-2α plays crucial roles in the regulation of mucosal epithelial model functions in the future.

**Supplementary Information:**

The online version contains supplementary material available at 10.1186/s12860-024-00509-5.

## Background

Sepsis, a life-threatening type of organ dysfunction caused by a maladjusted response to infection [1], is a recognized global health problem with a high mortality rate ranging between 30 and 50%. Although comprehensive therapies, including anti-inflammatory and anticoagulant applications, have been used clinically for several years. Due to its narrow window, each hour of delay in providing the right treatment results in a 7.6% drop in survival for sepsis patients. Therefore, early and systematic diagnosis is a vital task and it is urgent to strengthen the current knowledge of sepsis pathophysiology in order to develop new clinical treatment methods.

The intestinal tract may be the initiator of multiple organ dysfunction (MODS), which may be due to the destruction of intestinal barrier and subsequent translocation of intestinal bacteria [2, 3]. The integrity of the structure and function of intestinal epithelial cells is crucial in the intestinal barrier. Abnormal intestinal epithelial cells are often observed in patients with sepsis, which is considered to play an important role in bacterial translocation and systemic infection [4].

Studies have shown that under physiological conditions, intestinal epithelial cells are in a “physiological hypoxia” environment and that intestinal epithelial cells can adapt to hypoxia [5]. Under pathological conditions, small fluctuations in blood flow can cause a large decrease in oxygen transport, leading to intestinal mucosal ischaemia/hypoxia. In sepsis, during the inflammatory process, tissue metabolism is high, oxygen consumption increases, intestinal mucosal vessels constrict, mucosal permeability increases [6], oxygen supply decreases, and the degree of hypoxia in intestinal epithelial cells increases [7]. Hypoxia-inducible factor−1 is a key transcription factor required for the human body to adapt to hypoxic environments and plays an important role in the development of sepsis [8].

HIF−1 is composed of an oxygen-dependent α subunit and a constitutively expressed β subunit [9]. HIF−1α plays a key role in the acute hypoxia response [10]. Our recent study found that the expression of HIF−1α in the intestinal mucosa of rats with sepsis was upregulated and that the administration of the HIF−1α activator dimethyloxallyl glycine (DMOG) inhibited sepsis-induced inflammatory responses and oxidative stress levels, increased antioxidant levels, and alleviated intestinal mucosal barrier injury. The effect of the HIF−1α inhibitor BAY 87−2243 on the intestinal mucosal barrier was the opposite. These results suggest that HIF−1α has a protective effect against intestinal mucosal injury due to sepsis [11]. The mechanistic target of rapamycin/70-kDa ribosomal protein S6 kinase (mTOR/P70S6K) signalling pathway is an upstream regulator of HIF−1α and a key pathway in metabolism and plays an important role in the processes of energy regulation and autophagy [12]. Studies have shown that activating mTOR signaling pathway can upregulate the expression of HIF−1α, increase glycolysis, lead to metabolic reprogramming and activate “training immunity” to improve inflammation; inhibition of the mTOR signaling pathway has the opposite effect [13]. Whether mTOR/P70S6K signaling pathway regulates the expression of HIF−1α in intestinal epithelial injury in sepsis remains to be further studied.

This study established an in vitro model of the intestinal mucosal epithelial model in sepsis using LPS stimulation to induce human colorectal adenocarcinoma cells (Caco−2) to investigate whether the mTOR/P70S6K signalling pathway regulates HIF−1α expression to attenuate sepsis-induced impairment of intestinal mucosal epithelial model function. This study provides an experimental basis for the mechanism and treatment of intestinal mucosal injury in sepsis.

## Materials and methods

### Materials and reagents

Caco-2 human colorectal adenocarcinoma cells were purchased from the Cell Bank of Type Culture Collection of the Chinese Academy of Sciences (Shanghai, China) in 2019. Fluorescein isothiocyanate-dextran (FD-4) and lipopolysaccharides (LPS, 2630, Sigma, USA); rabbit anti-rat ZO-1, HIF-1α and occludin polyclonal antibodies (Invitrogen, USA); claudin-1 and β-actin antibodies (Abcam, UK); mTOR, P-mTOR, P70S6K and P-P70S6K polyclonal antibodies (Cell Signaling Technology); horseradish peroxidase (HRP)-labeled goat anti-rabbit antibody (Jackson ImmunoResearch, USA); dimethyloxallyl glycine (DMOG), BAY 87-2243, rapamycin, and MHY1486 (MedChemExpress, USA); and fetal bovine serum (FBS), Minimum Essential Medium (MEM), penicillin/streptomycin, N-L-alanyl-L-glutamine, sodium pyruvate, nonessential amino acids, dimethyl sulfoxide (DMSO) and 0.25% trypsin-EDTA (Gibco, USA).

### Cell culture

After thawing, Caco-2 cells were cultured in MEM containing 10% FBS, 100 U/100 mg/ml penicillin and streptomycin, 1% sodium pyruvate, 1% glutamine, and 1% nonessential amino acids at 37 °C in a 5% CO_2_ incubator. The medium was changed every 2 days. Cells were seeded on 24-well at a density of 2 × 10^5^ cells/ml (400 µl per well), when they were in the logarithmic growth phase and growing well. Culture medium (600 µl) was added from the bottom side and it was changed 24 h after seeding. The cell culture medium was changed every other day for the following week and then every day thereafter. Cell growth was observed regularly. Transepithelial electrical resistance (TEER) was measured daily using a Millicell ERS-2 Epithelial Volt-Ohm Meter (MerckMillipore, USA). After the cells were cultured for approximately 21 days, the TEER significantly increased and remained stable after the cells formed an intact and dense monolayer, indicating that the intestinal mucosal epithelial model was basically formed.

In the experiment, LPS, DMOG, BAY 87-2243, rapamycin and MHY1487 were all dissolved in the cell culture medium.

### Confirmation of the optimal concentrations of LPS, activator and inhibitor

Caco-2 cells were exposed to different concentrations of LPS, DMOG (HIF-1α activator), BAY 87-2243 (HIF-1α inhibitor), rapamycin (mTOR inhibitor) and MHY1487 (mTOR activator), and the optimal concentration was selected based on the effect of each concentration on the viability of Caco-2 cells or the expression of HIF-1α.

### Establishment of a Caco-2 cell injury model and experimental grouping

Caco-2 cells were selected and the experiment was divided into 2 parts. Part I: untreated control group (CON), LPS-induced epithelial cell injury model group (LPS), LPS + DMOG (HIF-1α activator) group (L + D) and LPS + BAY 87-2243 (HIF-1α inhibitor) group (L + B); Part II: CON group, LPS group, LPS + rapamycin (mTOR inhibitor) group (L + R), LPS + MHY1487 (mTOR activator) group (L + M), LPS + rapamycin + DMOG (L + R + D), and LPS + MHY1487 + BAY 87-2243(L + M + B).

In the CON group, cells were cultured in normal medium. In the LPS group, cells were stimulated with 600 µg/ml LPS. In the L + D group, cells stimulated with LPS were treated with 10^4^ nM DMOG. In the L + B group, cells stimulated with LPS were treated with 10 nM BAY 87-2243. In the L + R group, cells stimulated with LPS were treated with 10 nM rapamycin. In the L + M group, cells stimulated with LPS were treated with 100 nM MHY1487. In the L + R + D group, cells stimulated with LPS and treated with 10 nM rapamycin were incubated with 10^4^ nM DMOG. In the L + M + B group, cells stimulated with LPS and treated with 100 nM MHY1487 were incubated with 10 nM BAY 87-2243.

Caco-2 cells in all groups were cultured in culture medium (same cell culture). Except the CON group, Caco-2 cells in other treatment groups were pretreated with LPS (final concentration 600 µg/ml) for 2 h. DMOG (final concentration 10^4^ nM), BAY 87-2243 (final concentration 10 nM), rapamycin (final concentration 10 nM) and MHY1487 (final concentration 100 nM) were added in one time and continued to culture for another 24 h.

### Measurement of the TEER of the monolayer epithelial model

The electrode was immersed in 70% alcohol for 15 min, air-dried for 15 s, and then placed in Hanks’ balanced salt solution (HBSS) at 37 °C for 15 min. The culture medium was removed from the culture plate. Then, 400 µl of prewarmed HBSS was added to each well of the upper chamber, and 600 µl of prewarmed HBSS was added to each well of the lower chamber. Solutions were equilibrated at 37 °C for 30 min in the incubator, debris on the cell surface was washed away, and HBSS was removed. Preheated HBSS was added again, and the transmembrane resistance value was measured by Millicell ERS − 2 epithelial volt-ohmmeter to evaluate the cell permeability. The above steps were repeated with a blank carrier to obtain the blank value.

$$\begin{array}{l}{\rm{TEER}}\,\left( {{\rm{\Omega }} \cdot {\rm{c}}{{\rm{m}}^{\rm{2}}}} \right)\,{\rm{ = }}\,\left( {{\rm{measured}}\,{\rm{resistance}}\,{\rm{value - blank}}\,{\rm{value}}} \right)\\\,\left( {\rm{\Omega }} \right)\,{\rm{ \times }}\,{\rm{monolayer}}\,{\rm{surface}}\,{\rm{area}}\,\left( {{\rm{c}}{{\rm{m}}^{\rm{2}}}} \right){\rm{.}}\end{array}$$

### Detection of fluorescein isothiocyanate–dextran (FD-4) permeability

A Caco-2 monolayer model was established. After the cellswere treated with the experimental factors, the culture medium was removed, and 400 µl of HBSS solution containing 1 mg/ml FD-4 was added to the upper chamber, avoiding light exposure. After incubation at 37 °C with 5% CO_2_ for 2 h, 100 µl of the solution from the lower chamber was collected and measured using a fluorescence microplate reader (SPARK®, USA). The excitation wavelength was 490 nm, and the emission wavelength was 530 nm. A standard curve for the concentration was plotted, and the concentration of each sample was calculated based on the standard curve.

### Detection of TJs and HIF-1α protein expression by western blotting (WB)

After the cells in each experimental group were treated based on the predetermined experimental protocol, the cells were washed twice with cold phosphate buffered saline (PBS) and lysed with radioimmunoprecipitation assay (RIPA) buffer containing phenylmethylsulfonyl fluoride (PMSF). The cells were lysed on ice for 30 min. The supernatant was collected after centrifugation at 12,000 g for 15 min. After quantification using the bicinchoninic acid (BCA) assay, 40 mg of total protein was mixed with an equal volume of 2× loading buffer, boiled at 100 °C for 3–5 min to denature, and loaded onto a 6-10% SDS polyacrylamide gel for electrophoresis. The protein bands were transferred to a membrane and blocked with Tris buffered saline with Tween 20 (TBST) containing 5% bovine serum albumin (BSA) for 2 h at room temperature. TBST was used to wash the membrane 3 times (5 min per wash). Membranes were incubated with anti-HIF-1α (1:1000), anti-ZO-1 (1:1000), anti-occludin (1:1000), anti-claudin-1 (1:1000), anti-β-actin (1:2000), anti-p-mTOR (1:1000), anti-mTOR (1:1000), anti-p-P70S6K (1:1000), anti-P70S6K (1:1000), and anti-GAPDH (1:1000) at 4 °C overnight. After thorough washing with TBST, HRP-labeled goat anti-rabbit antibody was added to the membrane, and the membrane was incubated at room temperature for 2 h and washed 3 times with TBST. Protein signals were visualized using an enhanced chemiluminescence (ECL) kit (Beyotime Biotechnology, China). The membrane was photographed in a gel imaging system (ChemiDoc XRS + System, Bio-Rad Company, USA), and the greyscale value was analysed using Image J software (Bio-Rad Company, USA).

### Cell counting Kit-8 (CCK-8) assay

Caco-2 cells (70-80% confluent) were digested with trypsin and collected, followed by centrifugation to prepare a cell suspension. Then, 100 µl of cell suspension was added to a 96-well plate at a cell density of 2 × 10^4^ cells/ml. After incubation for 24 h (37 °C, 5% CO_2_), different groups were established, with 6 replicate wells for each group. After 24 h of incubation (37 °C, 5% CO_2_), 10 µl of CCK-8 solution was added to each well, followed by incubation (37 °C, 5% CO_2_) for 2 h. The absorbance was measured at 450 nm using a microplate reader (SpectraMax Plus 384, Meigu Molecular Instruments (Shanghai) Co., LTD, China). Cell viability is calculated according to the following formula:

$${\rm{Cell}}\,{\rm{viability}}\,{\rm{ = }}\,\left[ {\left( {{\rm{As - Ab}}} \right)\,{\rm{/}}\,\left( {{\rm{Ac - Ab}}} \right)} \right]\,{\rm{ \times }}\,{\rm{100\% }}$$

As: OD of the experimental hole (including cells, culture medium, CCK-8 solution and drugs); Ac: OD of the control hole (including cells, culture media, CCK-8 solution, without drugs); Ab: OD of blank hole (including culture medium, CCK-8 solution, excluding cells and drugs).

### Statistical analysis

Statistical analysis was performed using GraphPad Prism (Version 7.0, California, USA) statistical software. Measurement data conforming to normal distribution were presented as mean ± standard deviation ($\stackrel{-}{\text{x}}$±SD). One-way analysis of variance was used to analyze the differences among groups. For data with homogeneous variance, the least significant difference (LSD) test was used for pairwise comparisons. For data heterogeneous variance, Dunnett’s T3 test was used. While measurement data of skewed distribution were expressed as M (P25, P75), and the data among groups were tested via the nonparametric test. *P* < 0.05 was considered statistically significant.

## Results

### Confirmation of the optimal concentrations of LPS, DMOG and BAY 87-2243

**Fig. 1:**
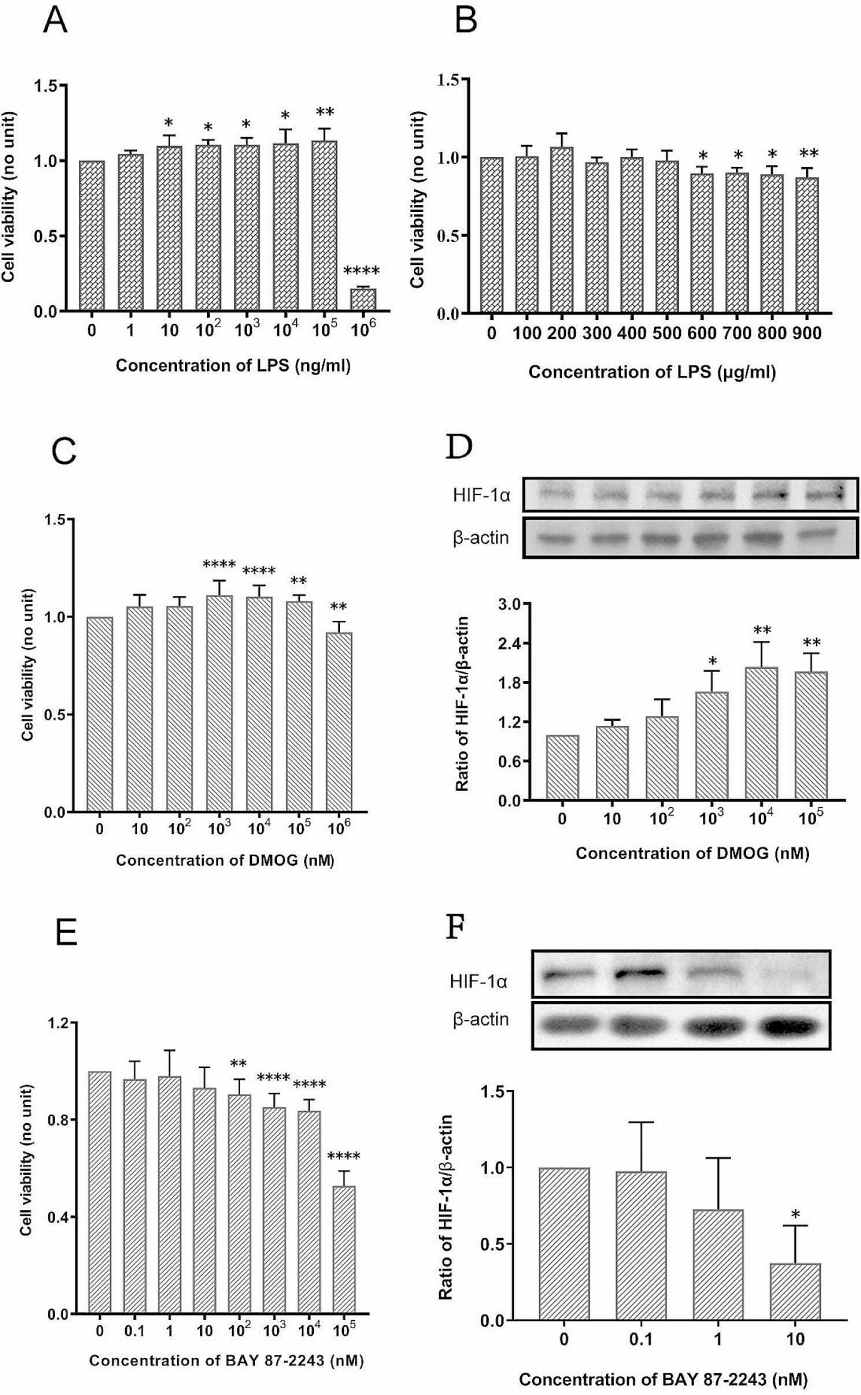
Confirmation of the optimal concentrations of LPS, DMOG and BAY 87-2243 for Caco-2 cells. (**A-B**) Effect of different concentrations of LPS on the viability of Caco-2 cells and the optimal concentration of LPS for this study was 600ug/ml. (**C**) Effect of different concentrations of DMOG on the viability of Caco-2 cells. (**D**) Effect of different concentrations of DMOG on the expression of HIF-1α in Caco-2 cells. (**E**) Effect of different concentrations of BAY 87-2243 on the viability of Caco-2 cells. (**F**) Effect of different concentrations of BAY 87-2243 on the expression of HIF-1α in Caco-2 cells. Optimal concentrations for DMOG and BAY 87-2243 were 10^4^ nM and 10 nM respectively. ^*^P < 0.05; ^**^P < 0.01; ^***^P < 0.001; ^****^P < 0.0001 vs. 0 nM

Different concentrations of LPS (0, 1, 10, 10^2^, 10^3^, 10^4^, 10^5^, 10^6^ ng/ml) were added to the Caco-2 cell culture medium. Compared with LPS free group (LPS 0 ng/ml), 10 ng/ml, 10^2^ ng/ml, 10^3^ ng/ml, 10^4^ ng/ml and 10^5^ ng/ml of LPS significantly increased cell viability, while 10^6^ ng/ml LPS significantly inhibited cell viability (Fig. 1A). Then LPS at 0, 100, 200, 300, 400, 500, 600, 700, 800, and 900 µg/ml were tested again, compared with LPS free, more than 600 µg/ml of LPS showed significant inhibitory effect on Caco-2 cell viability (Fig. 1B). Therefore, in this study, the optimal stimulating concentration was 600 µg/ml LPS. Caco-2 cells were exposed to different concentrations of DMOG and BAY 87-2243. DMOG (10^6^ nM) inhibited the viability of Caco-2 cells, and 10^3^, 10^4^, and 10^5^ nM DMOG significantly increased cell viability (Fig. 1C). BAY 87-2243 (10^2^ nM) had a significant inhibitory effect on Caco-2 cell viability, and BAY 87-2243 concentrations < 10 nM had no significant inhibitory effect on cell viability (Fig. 1E).

Different DMOG concentrations were evaluated within the range of concentrations that did not inhibit the viability of Caco-2 cells. WB detection results indicated that 10^4^ nM DMOG most significantly up regulated HIF-1α expression (Fig. 1D) and that 10 nM BAY22-8742 effectively inhibited HIF-1α expression (Fig. 1F). Based on the results of CCK experiment and WB detection, the optimal concentrations of DMOG and BAY87-2243 were 10^4^ nM and 10 nM, respectively.

### Confirmation of the optimal concentrations of mTOR activators and inhibitors

**Fig. 2:**
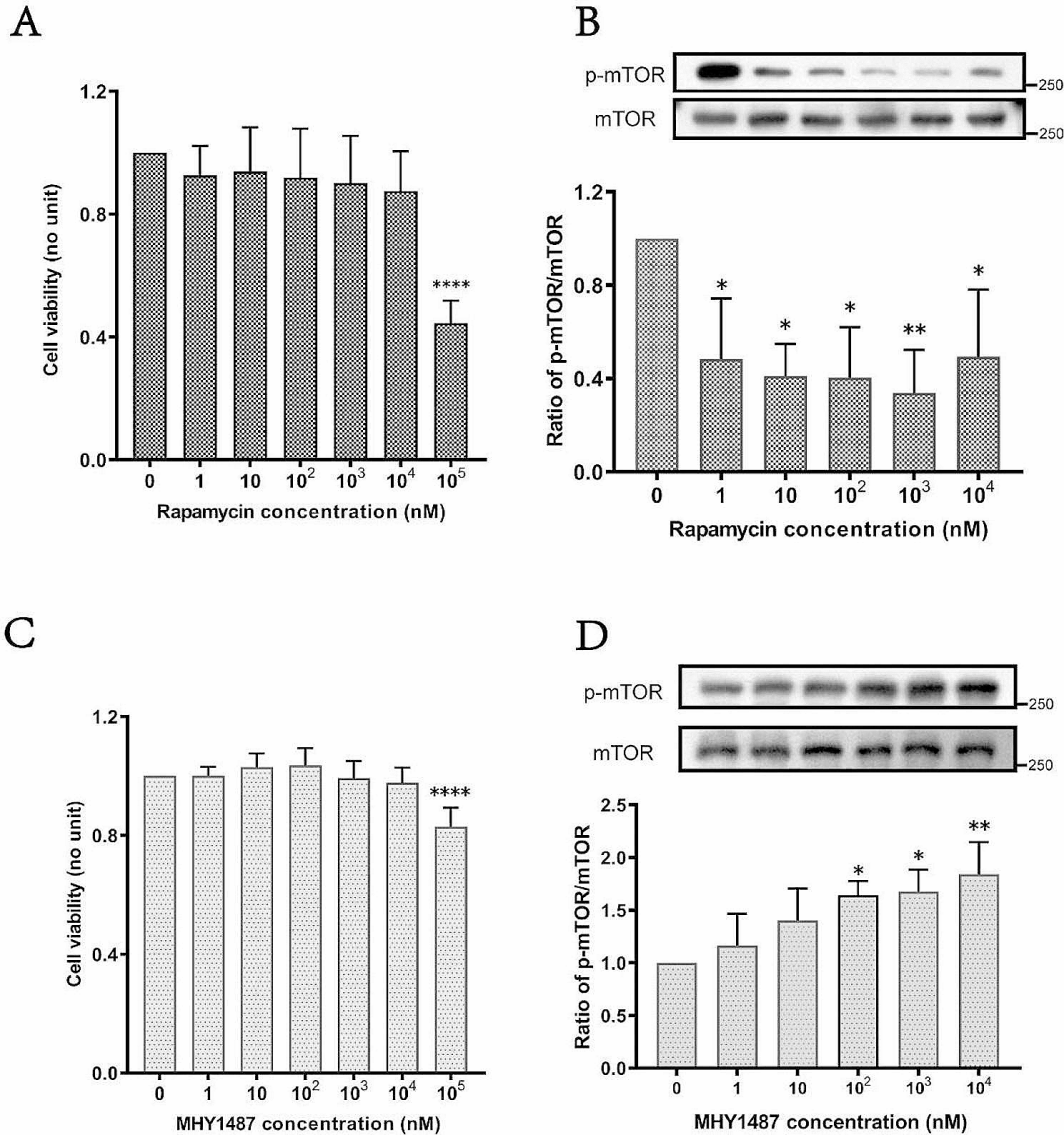
Confirmation of the optimal concentrations of rapamycin and MHY1487 for Caco-2 cells. (**A**) Effect of different concentrations of rapamycin on the viability of Caco-2 cells. (**B**) Effect of different concentrations of rapamycin on the expression of p-mTOR in Caco-2 cells. (**C**) Effect of different concentrations of MHY1487 on the viability of Caco-2 cells. (**D**) Effect of different concentrations of MHY1487 on the expression of p-mTOR in Caco-2 cells. Optimal concentrations for rapamycin and MHY1487 were 10 nM and 100 nM, respectively. ^*^P < 0.05; ^**^P < 0.01; ^****^P < 0.0001. vs. 0 nM

The results of the CCK-8 assay indicated that 10^5^ nM rapamycin significantly inhibited the viability of Caco-2 cells, while concentrations of 10^4^ nM and lower had no effect on the viability of Caco-2 cells (Fig. 2A). When 0 ∼ 10^4^ nM rapamycin was added, WB indicated that 1 nM rapamycin had the most significant inhibitory effect on p-mTOR/mTOR expression (Fig. 2B). MHY1487 (10^5^ nM) significantly inhibited the viability of Caco-2 cells, while MHY1487 at concentrations ≤ 10^4^ nM had no significant effect on the viability of Caco-2 cells (Fig. 2C). WB detection indicated that 10^4^ nM MHY1487 promoted the most significant upregulation of p-mTOR/mTOR expression (Fig. 2D, *P* < 0.05). Based on the results of CCK experiment and WB detection, the optimal concentrations of rapamycin and MHY1487 were 10 nM and 100 nM, respectively.

### Effect of HIF-1α activator and inhibitor on the TEER and FD-4 concentration of the Caco-2 cell epithelial model after LPS stimulation

**Fig. 3:**
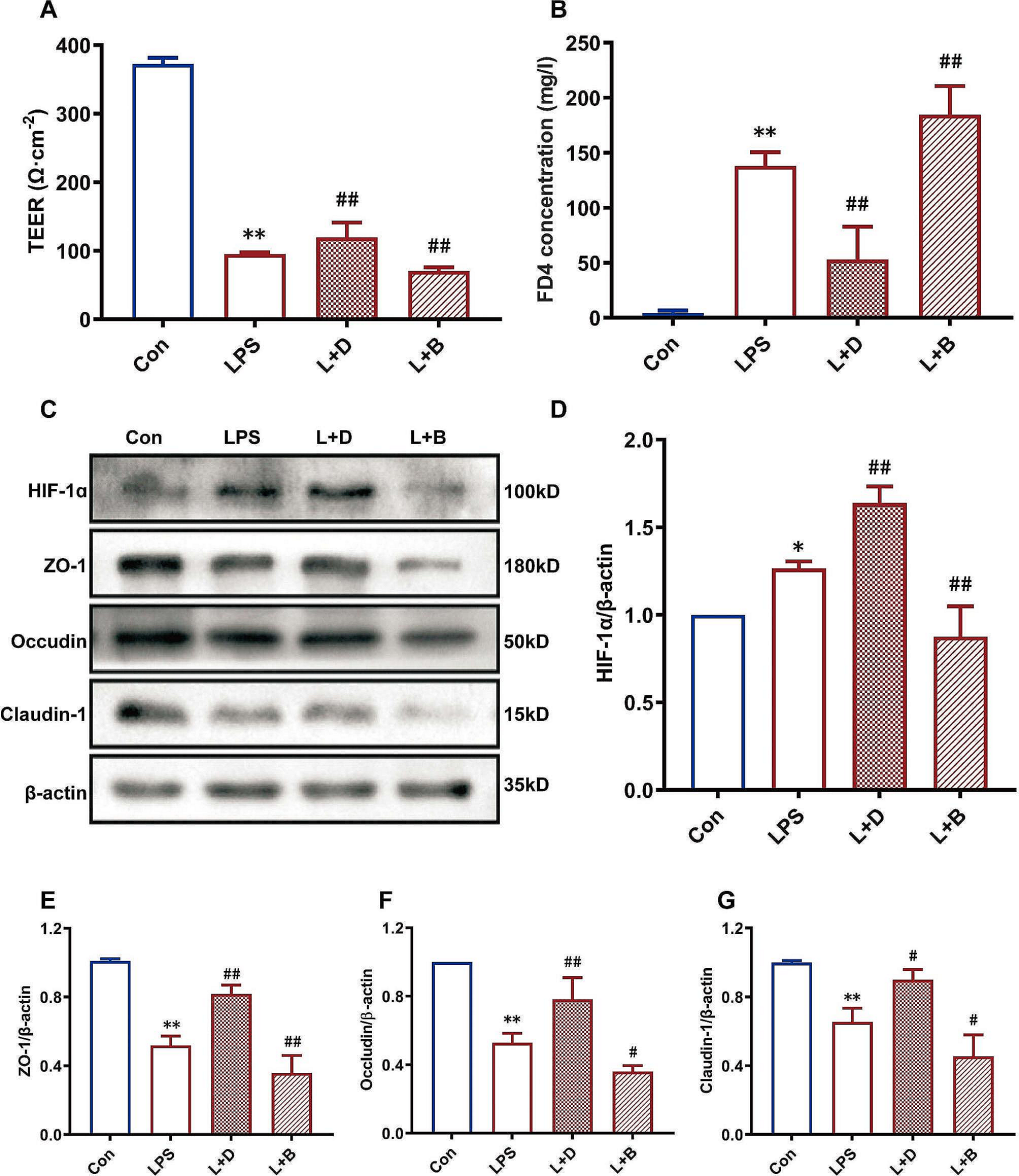
Effects of HIF-1α activators and inhibitors on the TEER (**A**), FD-4 concentration (**B**), and the expression of HIF-1α (**C, D**) and TJ-related proteins (**C, E-G**) in the Caco-2 cell epithelial model after LPS stimulation. LPS-induced damage to Caco-2 cell epithelial model showed the decrease of TEER value, the increase of FD4 concentration and down-regulation of TJ-related proteins. DMOG, the HIF-1α activator, alleviated the LPS-induced damage to Caco-2 cell epithelial model, increased TEER value, decreased FD4 concentration and up-regulated the expression of TJ-related proteins. BAY 87-2243, a HIF-1α inhibitor, had the opposite effect. LPS up-regulated HIF-1α expression, DMOG further up-regulated HIF-1α expression, while BAY 87-2243 had the opposite effect Con: control group, LPS: LPS group, L + D: LPS + DMOG group, L + B: LPS + BAY 87-2243 group. *P < 0. 05, **P < 0. 01 vs. Con group; #*P* < 0.05, #*P* < 0.01 vs. LPS group

After LPS stimulation, the TEER of Caco-2 cells was significantly lower than that of the control group (*P* < 0.05). Compared with LPS alone, DMOG significantly increased the TEER (*P* < 0.05) (Fig. 3A) and decreased the FD-4 concentration (*P* < 0.05) (Fig. 3B); the opposite effect was observed for BAY 87-2243 (Fig. 3A, B).

### Effects of HIF-1α activators and inhibitors on TJ protein expression in Caco-2 cells after LPS stimulation

WB indicated that LPS increased the protein expression level of HIF-1α in Caco-2 cells (*P* < 0.05; Fig. 3C and D). Compared with LPS alone, DMOG upregulated the expression of HIF-1α and the TJ-related proteins ZO-1, occludin and claudin-1 (*P* < 0.05); the opposite effect was observed for BAY 87-2243 (*P* < 0.05), as shown in Fig. 3C, E-G.

### The attenuation of LPS-induced epithelial model injury in Caco-2 cells by HIF-1α is regulated by the mTOR/P70S6K pathway

**Fig. 4:**
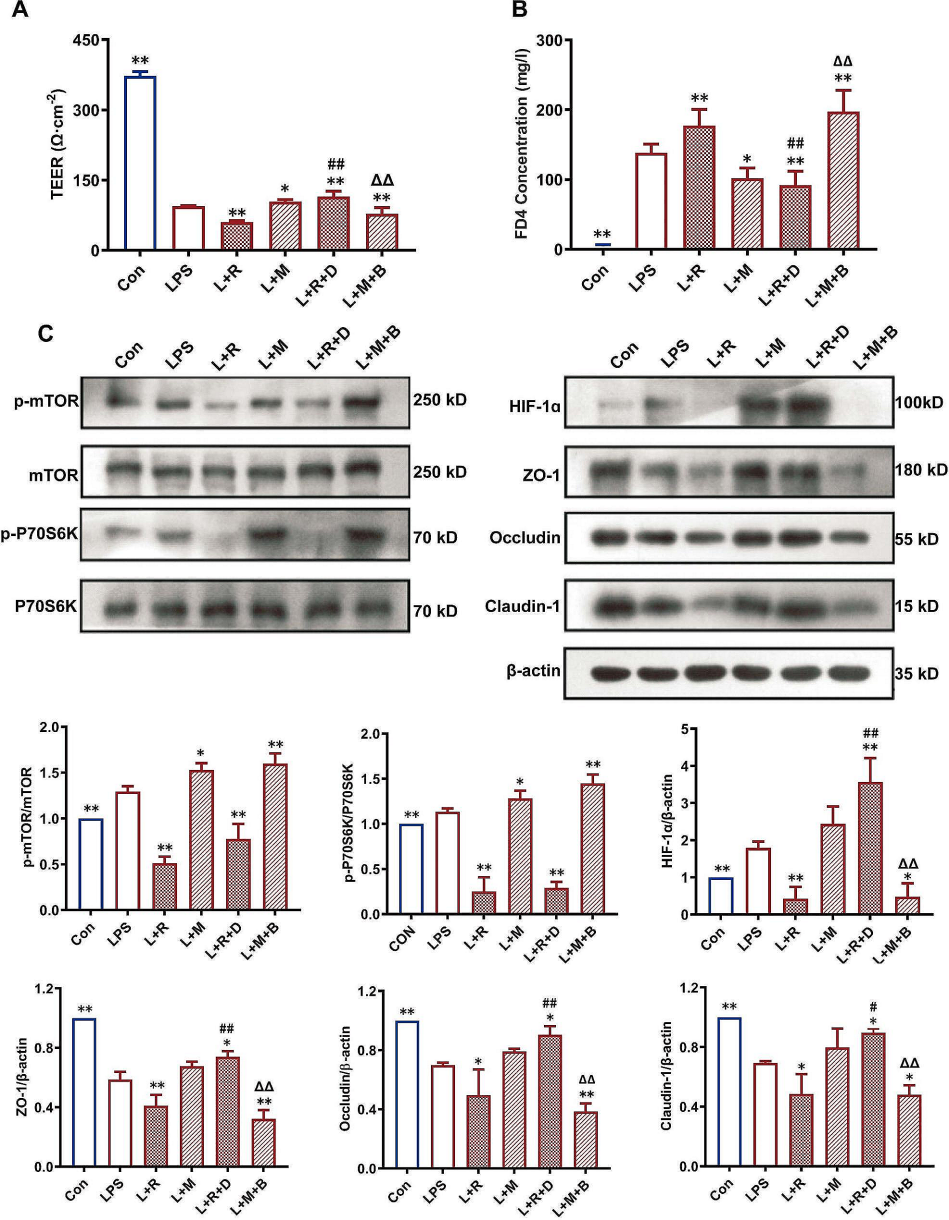
The effect of HIF-1α on LPS-induced epithelial model damage to Caco-2 cells is regulated by the mTOR/P70S6K pathway. (**A**) Rapamycin (mTOR inhibitor) decreased TEER of Caco-2 cell monolayer, and MHY1487 (mTOR activator) increased TEER of Caco-2 cell monolayer. DMOG (HIF-1α activator) can rescue the decrease of TEER caused by rapamycin, and BAY87-2243 (HIF-1α inhibitor) can rescue the increase of TEER caused by MHY1487; (**B**) Rapamycin increased the FD4 concentration in Caco-2 cell monolayer, while MHY1487 had opposite effect. DMOG rescued the increase in FD4 concentration caused by rapamycin, and BAY 87-2243 rescued the decrease in FD4 concentration caused by MHY1487; (**C**) Western blot was used to detect the expression of p-mTOR, mTOR, p-P70S6K, P70S6K, HIF-1α and TJs (ZO-1, occludin and claudin-1) Con: control group, LPS: LPS group, L + R: LPS + rapamycin group, L + M: LPS + MHY1487 group, L + R + D: LPS + rapamycin + DMOG group. L + M + B: LPS + MHY1487 + BAY 87-2243 group. *P < 0. 05, **P < 0. 01 vs. LPS group; ##*P* < 0. 01 vs. L + R group; △*P* < 0. 05, △△*P* < 0. 01 vs. L + M group

Compared with those in the LPS group, the TEER of the Caco-2 cell epithelial model in the L + R group significantly decreased, the FD-4 concentration significantly increased (Fig. 4A-B), the expression levels of p-mTOR/mTOR, p-P70S6K/P70S6K and HIF-1α significantly decreased, and the expression levels of TJ-related proteins decreased (Fig. 4C. In the L + M group, the TEER of the Caco-2 cell epithelial model significantly increased, and the FD-4 concentration significantly decreased (Fig. 4A-B, *P* < 0.05). The expression levels of p-mTOR/mTOR and p-P70S6K/P70S6K significantly increased (*P* < 0.05), while the expression levels of HIF-1α and TJ-related proteins did not significantly increase (*P* > 0.05, Fig. 4C). The results indicated that compared with those in the L + R group, the TEER in the L + R + D group significantly increased, the FD-4 concentration significantly decreased (*P* < 0.05, Fig. 4A-B), and the expression levels of p-mTOR/mTOR and p-P70S6K/P70S6K did not change significantly (Fig. 4C). The expression levels of HIF-1α and TJ-related proteins increased (*P* < 0.05, Fig. 4C). Compared with those in the L + M group, the TEER of Caco-2 cells in the L + M + B group significantly decreased (*P* < 0.05), the FD-4 concentration significantly increased (*P* < 0.05, Fig. 4A-B), and the expression levels of p-mTOR/mTOR and p-P70S6K/P70S6K did not change significantly (*P* > 0.05, Fig. 4C). The expression levels of HIF-1α and TJ-related proteins decreased (*P* < 0.05, Fig. 4C).

## Discussion

Using Caco−2 human colorectal adenocarcinoma cells, we showed that HIF1-alpha may alleviate LPS-induced intestinal epithelial model in vitro.HIF−1α attenuated LPS-induced Caco−2 cell injury by upregulating structural protein expression and decreasing permeability; additionally, HIF−1α alleviated LPS-induced Caco−2 injury, a process that might be regulated by mTOR/P70S6K signalling.

LPS is a major component of the outer membrane of gram-negative bacteria, which can activate cells such as macrophages, endothelial and epithelial cells, and enable host cells to produce cytokines and inflammatory mediators [11]. The inflammatory response is a defense mechanism against infection. In fact, systemic inflammatory responses, such as sepsis, can lead to multiple organ failure or death [1]. Sepsis is a clinical syndrome characterized by systemic inflammation and circulatory system damage caused by infection [1, 14]. LPS plays a crucial role in causing intestinal and systemic inflammatory responses [1]. Previous studies have shown that LPS increases intestinal TJ permeability and destroys the intestinal barrier [15, 16].

Previous studies have shown that LPS regulate inflammation and the immune system by activating signaling pathways mediated by relevant receptors [17]. A high dose of LPS induces a strong inflammatory response, leading to sepsis or septic shock. On the contrary, a low dose of LPS induces a protective cross-tolerance state [18]. Calvello et al.demonstrated that 1 µg/ml of LPS can trigger an inflammatory response in Caco−2 cells, but does not induce cell death [19]. Endotoxin tolerance has been shown to prevent lethality after ischemia/reperfusion injuries, sepsis, and endotoxic shock. One study showed that lethal propionibacterium acnes (PA)-primed LPS-induced hepatic injury can be prevented by administering a tolerizing dose of LPS prior to PA-priming [20]. Another study [21] demonstrated that LPS reprocessing may achieve neuroprotective effects by regulating inflammatory mediators, inducing neuroprotective factors and inhibiting the release of proinflammatory cytokines in rat models of cerebral ischemic injury. In our present study, we found that LPS below 10^5^ ng/ml increased Caco−2 activity, and this concentration was many times higher than that previously reported ones, which may be related to different reagent manufacturers and models. The exact reasons need further study. As a classical model for in vitro simulation of the intestinal epithelial model and drug transport, the Caco−2 monolayer epithelial model has been widely used. Another study, which [22] successfully established an organoid intestinal injury model suitable for sepsis, examined the effects of LPS-induced intestinal damage similar to sepsis. The biological characteristics of this model were systematically evaluated, and the results revealed that this organoid-based model provides a platform for revealing the potential mechanism of sepsis associated intestinal epithelial injury and screening therapeutic agents.

FD−4 is a marker of changes in intestinal permeability and the intestinal barrier [23]. TJ-related proteins, including ZO−1, occludin, and claudin−1, play important roles in maintaining intercellular connections and cell barriers [24, 25]. Occludin plays an important role in maintaining the intestinal TEER, has adhesion functions, and serves as a fence. Claudin−1 affects the permeability of intercellular substances, especially cations, forming a selective paracellular ion channel. ZO−1 binds to a variety of cytoskeletal proteins and plays a supporting role in TJs [24, 25].

Destruction of the intestinal barrier was exacerbated in intestinal HIF−1α-deficient mice [26]. Colgan et al. [27] showed that compared with wild-type animals, mice with intestinal epithelial HIF−1α mutations (inhibition of HIF−1α expression) had more severe colitis, weight loss, reduced colon length, and increased intestinal permeability. By contrast, intestinal epithelium with the von Hippel-Lindau mutation (which causes the sustained expression of HIF−1α) has a protective effect on the intestine. Claudin−1 has been shown to regulate dysfunction in various human diseases [28]. Singer et al. [1] confirmed that HIF−1α has a crucial regulatory role with respect to claudin−1 in the intestinal epithelium, and through knock-out (KO) and overexpression experiments, it was shown that HIF−1α plays a fundamental regulatory role in the expression of claudin−1 at the gene promoter level. Reintroduction of the claudin−1 gene into HIF−1β KO cells can lead to abnormal cell barrier function and morphology. In TJ target screening, claudin−1 is the main cause of morphological abnormalities in HIF−1α-deficient intestinal epithelial cell lines [29, 30].

In this study, HIF−1α simulator agonists or inhibitors was used with LPS in vitro. HIF−1α activation and permeability of Caco−2 cells decreased subsequently by up-regulating the expression of TJS; on the contrary, the permeability of Caco−2 cells increased after HIF−1α inhibition by down-regulating the expression of TJs. These results further confirmed that HIF−1α has protective effects against LPS-induced intestinal mucosal epithelial model injury through regulating the expression of TJs and our conclusion was consistent with our previous animal research [9].

Study also demonstrated that the mTOR signalling pathway is the main sensor that responds to the energy status and autophagy in energy consumption [31] and has a cascade amplification effect on HIF−1α [32, 33]. The mTOR signalling pathway may play an important role in intestinal dysfunction in sepsis. mTOR, a part of mammalian target of rapamycin complex 1 (mTORC1), is activated in the form of phosphorylation, p-mTOR. Protein kinase B (AKT) is activated downstream by phosphatidylinositol−3-kinase (PI3K) and subsequently activates mTOR [31]. p-mTOR can phosphorylate ribosomal protein S6 kinase (p70S6K) [32, 33]; subsequently, p70S6K activates rpS6 to promote HIF−1α protein synthesis [34, 35]. On the contrary, p-mTOR can also phosphorylate eukaryotic translation initiation factor 4E-binding protein 1 (4E-BP1) [36], which disrupts 4E-BP1 binding to eukaryotic translation inhibitory factor 4E (eIF−4E) and then inhibits eIF−4E [37], thereby blocking HIF−1α protein synthesis [38]. mTOR inhibitors can inhibit HIF−1α protein synthesis and reduce HIF−1α activity [39]. In septic rats, rapamycin can significantly reduce the levels of p-mTOR, p-P70S6K and HIF−1α in the myocardium and exert a protective effect by inhibiting autophagy in cardiomyocytes [40].

To further ascertain the crosstalk between the mTOR/70S6K signaling pathway and HIF−1α protein synthesis. Firstly, mTOR activator/inhibitor was used with LPS treatment on Caco−2. Consequently, mTOR inhibition, p-mTOR/mTOR and p-P70S6K/P70S6K ratio decreased and the downstream molecule HIF−1α expression was down-regulated, which counteracted the protective effect of HIF−1α on LPS-induced intestinal mucosal cell injury, while the mTOR activator partially alleviated LPS-induced intestinal mucosal injury, a process that may not act through HIF−1α. The specific mechanism needs to be further clarified. Secondly, when HIF−1α activator and the mTOR jointly acted on LPS-induced intestinal mucosal injury, The results showed that DMOG could partially alleviate the negative effect of rapamycin on intestinal mucosal injury, while the HIF−1α inhibitor and the mTOR activator jointly acted on LPS-induced intestinal mucosal injury. These results further confirmed HIF−1α has a protective effect on LPS-induced intestinal mucosal cell injury, a process that is regulated by the mTOR/P70S6K signalling pathway.

There are some limitations in this study. LPS is the endotoxin on the cell membrane of Gram-negative bacteria, the main pathogen of intestinal sepsis, and is considered the prototype of pathogen-associated molecular patterns (PAMPS) [41]. Toll-like receptors (TLRs) are the pattern recognition receptors (PRRs) that recognize PAMPs in microbial species, including bacterial flagellin recognized by TLR5. Of all PRRs in innate immunity, Toll-like receptor 2 (TLR2) recognizes the structurally broadest range of different bacterial PAMPs, because of its biomedical importance and because its genetics and biochemistry are presently most completely known among all Gram-positive bacteria. Initial reports indicated that TLR2 binds peptidoglycan (PG), but the effects were later found to be due to contaminating highly active natural lipoproteins and/or lipopeptides of PAMPs) [42]. In contrast, peptidoglycan is recognized by nucleotide-binding oligomerization domains 1 and 2 (NOD1 and NOD2) [43]. Thus, it would be helpful to use other PAMPs for example for gram-positive bacteria to compare the effect in future studies.

In this study, we investigated the protective effect of the mTOR/HIF−1α pathway on intestinal mucosal epithelial model injury in sepsis by detecting the pathological structure of the intestinal mucosa, tight junction proteins, and resistance and permeability of the intestinal mucosal epithelial model. The effect of the mTOR/HIF−1α pathway on the ultrastructural changes in the intestinal mucosal epithelial model induced by sepsis needs to be further explored. Previous studies have shown that HIF−1α directly induces the expression of many metabolic genes in cancer cells to enhance aerobic glycolysis, such as glucose transporters (GLUTs), pyruvate dehydrogenase kinase 1 (PDHK1), lactate dehydrogenase A (LDHA), to meet the energy needs of tumor growth [44]. Intestinal epithelial cell GLUT2 knockout mice or by inhibiting glucose metabolism can improve the damage to intestinal epithelial model function induced by hyperglycemia and inhibit intestinal bacterial ectopic [45]. Whether HIF−1ɑ induced by hypoxia in septic intestinal epithelial cells can provide energy for intestinal epithelial cells, and reduce mucosal damage by recruiting GLUT1 remains to be solved. This is also the shortcoming of the study.

Additionally, both DMOG and BAY 87−2243 are indirect regulators of HIF−1α expression. Further studies using HIF−1α gene knockout or overexpression technology could clarify the protective effect of the mTOR/HIF−1ɑ pathway on sepsis-induced intestinal mucosa injury. Previous studies have shown that the choice of experiments in normoxic [46] or hypoxic [47] environments should be based on the purpose of the experiment. In this study, the essence of sepsis-induced intestinal mucosal injury is intestinal mucosal ischemia and hypoxia; thus, the experiment was conducted under normal oxygen. In the future, we will challenge the low oxygen environment to explore the regulatory effect of HIF−1α on sepsis intestinal mucosal injury.

## Conclusions

In summary, the results from this study further verified that HIF-1α attenuated LPS-induced epithelial model dysfunction in intestinal epithelial cells (Caco-2) through a mechanism that may be regulated by the mTOR/P70S6K signalling pathway to improve changes in TJ structure and the expression of TJ-related proteins and to reduce intestinal mucosa permeability. This study provides the premise and basis for further research in the future.

### Electronic supplementary material

Below is the link to the electronic supplementary material.

Supplementary Material 1

## Data Availability

Supplementary material associated with this article can be found, in the online version.The email address of the corresponding author is yuh_li@zju.edu.cn.
