# Supplementary material for: mTOR signaling pathway regulation HIF-1 α effects on LPS induced intestinal mucosal epithelial model damage
Source: BMC Mol Cell Biol. 2024 Apr 23;25:13. doi: 10.1186/s12860-024-00509-5 (PMC11036631; doi:10.1186/s12860-024-00509-5)
Supplement: Supplementary file 1 — Supplementary Material 1 [file 12860_2024_509_MOESM1_ESM.pdf]

Fig 1A Effect of different concentrations of LPS (0~1000 μg/ml) on the viability of Caco-2 cells

0	0.001	0.01	0.1	1	10	100	1000
1.1107	1.1632	1.1538	1.2041	1.1657	1.1832	1.2427	0.2166
1.1216	1.1552	1.1383	1.2042	1.2052	1.1508	1.1499	0.1979
1.0252	1.1036	1.2076	1.134	1.0495	1.2673	1.235	0.2022
1.0596	1.083	1.2025	1.1458	1.1659	1.12	1.2607	0.1947
1.0554	1.0962	1.1519	1.2129	1.2276	1.2224	1.1567	0.21

Fig 1B Effect of different concentrations of LPS (0~900 μg/ml) on the viability of Caco-2 cells

0	100	200	300	400	500	600	700	800	900
1.1613	1.0397	1.1486	1.0893	1.089	1.1693	1.1204	1.0231	1.0162	0.9823
1.0577	1.1304	1.164	1.0182	1.1093	1.0524	0.9024	1.0034	0.9466	0.9769
1.1119	1.1507	1.2837	1.1104	1.1056	1.1239	0.9866	0.9631	0.9041	0.8731
1.1842	1.196	1.1408	1.1191	1.1433	1.0135	1.0832	1.0707	1.0868	1.0549
1.1306	1.1815	1.2877	1.1458	1.1812	1.1575	1.0465	1.0187	1.0751	1.062
1.0751	1.0531	1.1016	1.0345	1.0972	1.0672	0.9306	0.9937	0.9951	0.9318

Fig 1C Effect of different concentrations of DMOG on the viability of Caco-2 cells.

0	0.01	0.1	1	10	100	1000
1.5144	1.5237	1.5795	1.5715	1.6035	1.597	1.3255
1.4045	1.5583	1.4539	1.5601	1.572	1.517	1.2865
1.3927	1.5687	1.5616	1.7305	1.5953	1.5412	1.2128
1.3896	1.4993	1.51	1.5201	1.4657	1.5544	1.4179
1.333	1.3109	1.3813	1.5034	1.5712	1.4293	1.2656
1.3728	1.3787	1.3565	1.4201	1.4083	1.4189	1.2448
1.5144	1.5237	1.5795	1.5715	1.6035	1.597	1.3255
1.4045	1.5583	1.4539	1.5601	1.572	1.517	1.2865
1.3927	1.5687	1.5616	1.7305	1.5953	1.5412	1.2128
1.3896	1.4993	1.51	1.5201	1.4657	1.5544	1.4179
1.333	1.3109	1.3813	1.5034	1.5712	1.4293	1.2656
1.3728	1.3787	1.3565	1.4201	1.4083	1.4189	1.2448

Fig 1D Effect of different concentrations of DMOG on the expression of HIF-1α in Caco-2 cells.

0	10nM	100nM	1μM	10μM	100μM
1	1.12331722	1.54410752	2.01470367	2.47394865	2.23338098
1	1.23642423	1.2864107	1.5542506	1.84465905	1.98622992
1	1.05040485	1.0271827	1.41321155	1.78762214	1.68116451

Fig 1E Effect of different concentrations of BAY87-2243 on the viability of Caco-2 cells.

0	0.1	1	10	100	1	10	100
1.2593	1.1369	1.1833	1.0109	1.088	1.0833	1.0511	0.6873
1.3848	1.2965	1.4035	1.2496	1.3063	1.1499	1.2305	0.8664
1.4243	1.4499	1.4744	1.4072	1.318	1.1152	1.1853	0.8657
1.4855	1.4631	1.4771	1.464	1.3303	1.3477	1.3157	0.885
1.4469	1.475	1.5093	1.4066	1.3149	1.27	1.2538	0.8717
1.4338	1.2095	1.2316	1.114	1.1016	1.0479	1.0628	0.7155
1.3608	1.2066	1.2488	1.1971	1.1881	1.1777	1.1967	0.6221
1.5046	1.3841	1.1571	1.4522	1.3429	1.3451	1.1733	0.8114
1.5136	1.4645	1.4553	1.3942	1.3457	1.3111	1.2534	0.7972
1.4278	1.4795	1.5334	1.4112	1.3814	1.2477	1.2132	0.77
1.3208	1.4263	1.4948	1.3891	1.2853	1.2124	1.1467	0.745
1.2902	1.3248	1.348	1.2477	1.2796	1.1686	1.1299	0.565

Fig 1F Effect of different concentrations of BAY87-2243 on the expression of HIF-1α in Caco-2 cells

0	0.1	1	10
1	1.32924303	1.04040924	0.48923149
1	0.89340593	0.37451574	0.09312675
1	0.7053866	0.76685348	0.54126625

Fig 1D-HIF-1α

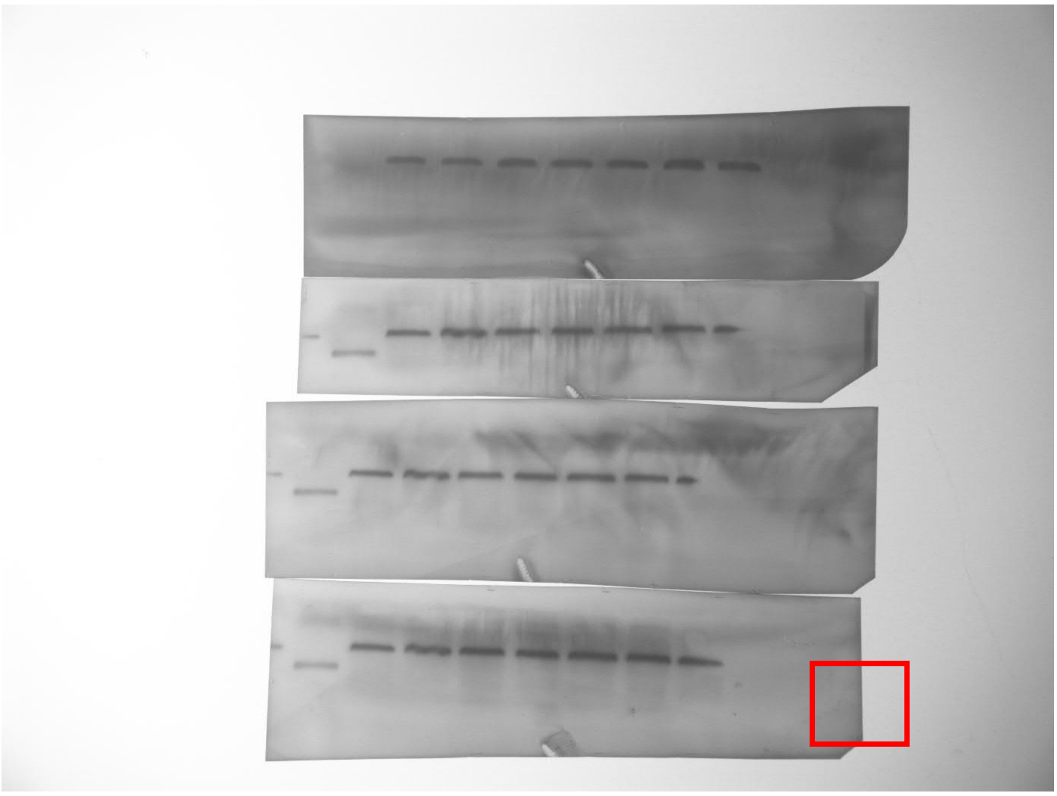

Fig 1D-β-actin

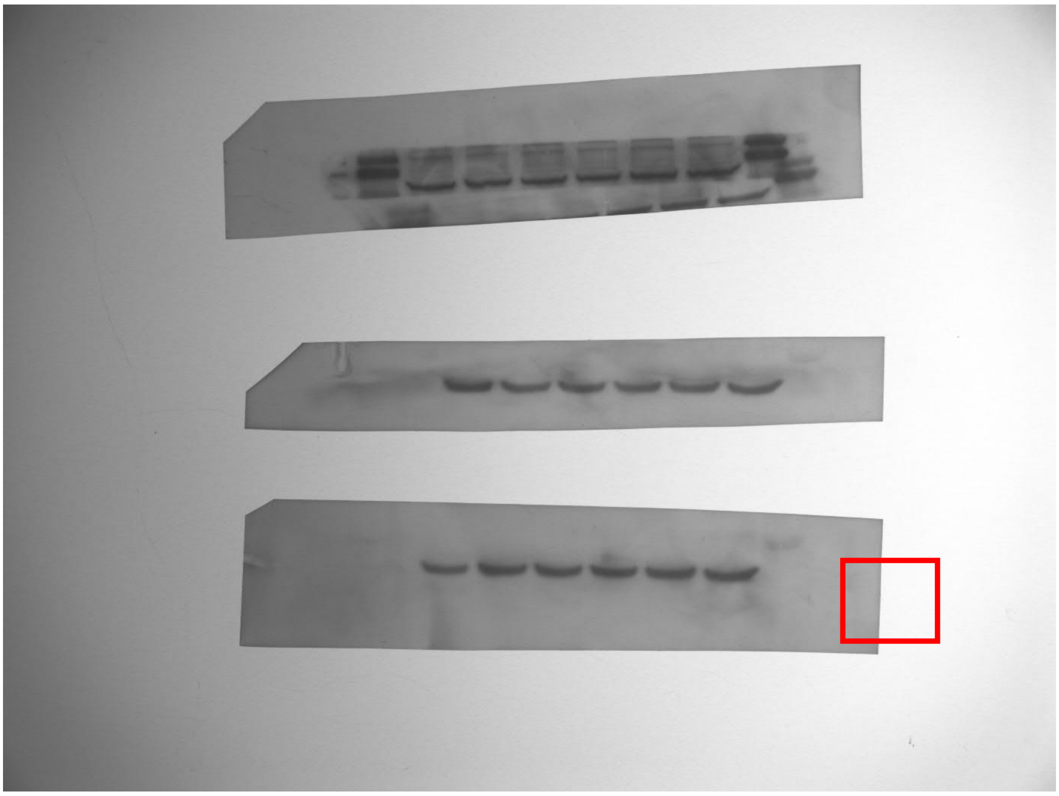

Fig 1F-HIF-1α

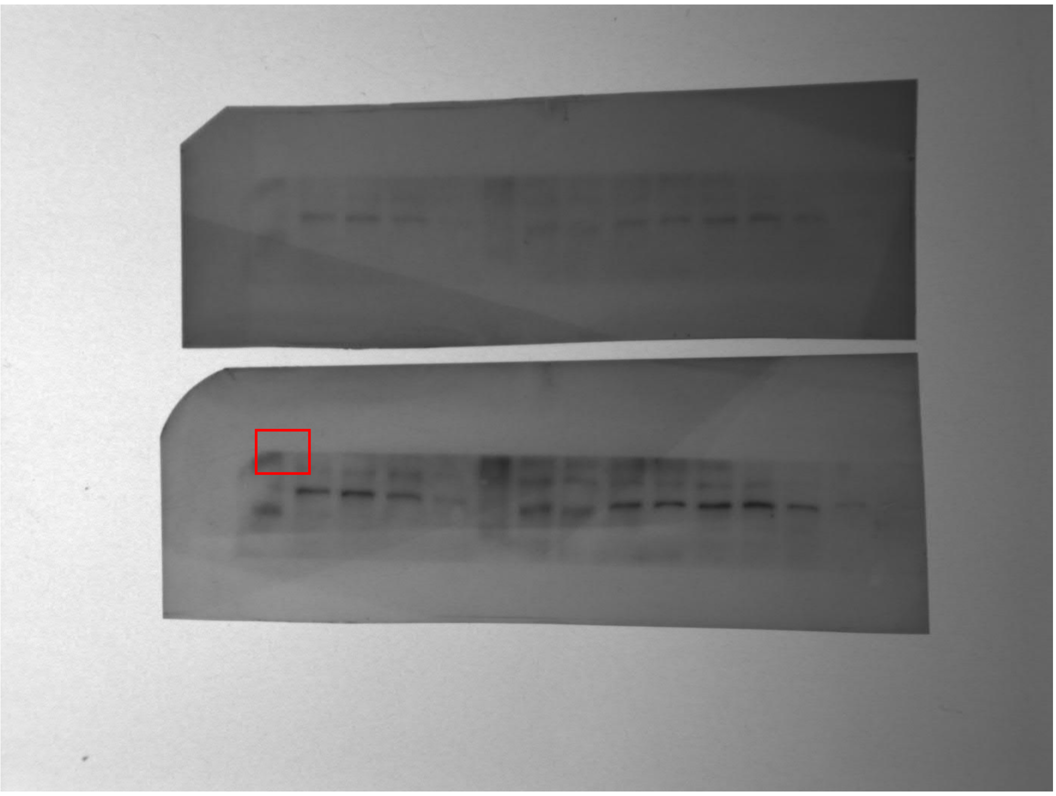

Fig 1F-β-actin

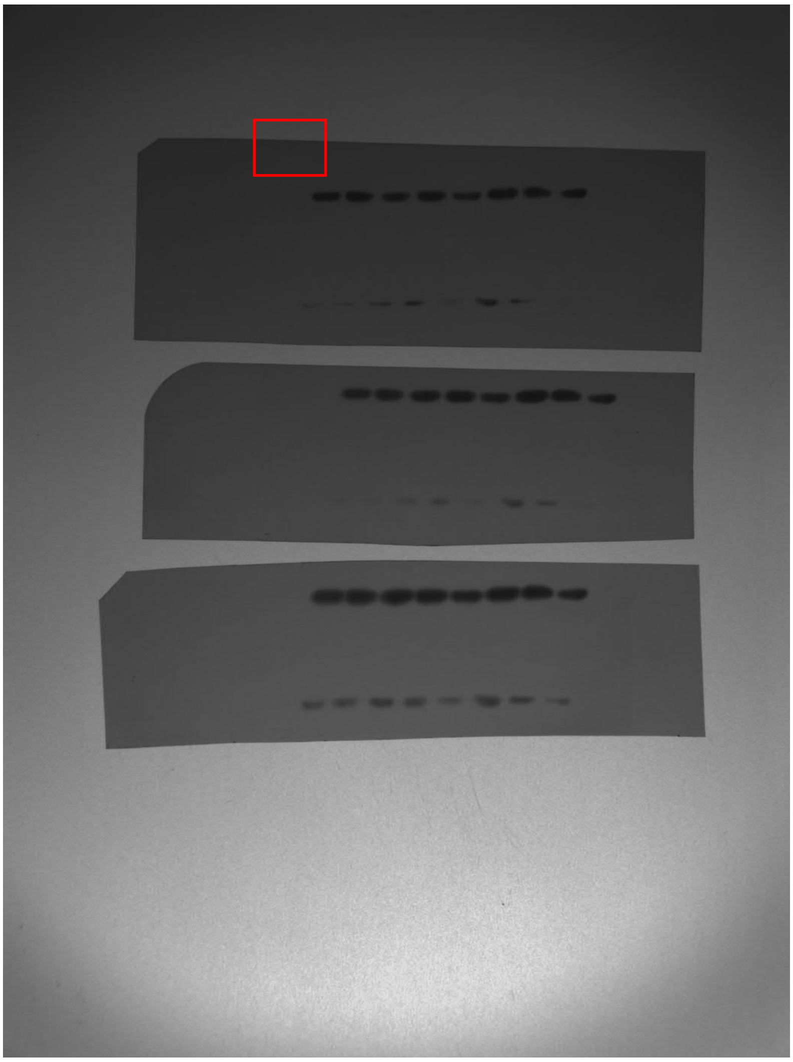

Fig 2A Effect of different concentrations of rapamycin on the viability of Caco-2 cells

0	0.001	0.01	0.1	1	10	100
1.3452	1.2628	1.2917	1.3094	1.2219	1.3429	0.6667
1.3298	1.3673	1.4887	1.3876	1.3415	1.1869	0.7266
1.3245	1.3546	1.3531	1.3222	1.3125	1.3282	0.7128
1.3097	1.3915	1.501	1.2993	1.3777	1.3518	0.7335
1.313	1.2449	1.2085	1.33	1.246	1.2552	0.6668
1.2565	1.2839	1.0838	1.2274	1.1646	1.1646	0.6094
1.2452	1.0623	1.0751	0.9135	1.0245	1.0418	0.5446
1.3098	1.1657	1.0804	1.0558	0.9871	1.033	0.5477
1.3245	1.0481	1.0376	1.0127	1.0211	0.9023	0.4823
1.3097	1.1625	1.0693	1.0061	0.964	1.0294	0.4735
1.313	1.0376	1.0612	0.9988	0.9617	0.878	0.5102
1.2565	1.1591	1.4668	1.5414	1.5266	1.2424	0.6194

Fig 2B Effect of different concentrations of rapamycin on the expression of p-mTOR in Caco-2 cells.

0	1nM	10nM	100nM	1μM	10μM
1	0.78057036	0.32354609	0.46022279	0.40077739	0.76755711
1	0.37344893	0.33827551	0.16609843	0.1328039	0.19438527
1	0.29716344	0.56914462	0.58739632	0.48299098	0.5181219

Fig 2C Effect of different concentrations of MHY1487 on the viability of Caco-2 cells.

0	0.001	0.01	0.1	1	10	100
1.4839	1.461	1.4832	1.6352	1.415	1.3696	1.1823
1.4694	1.472	1.5399	1.6166	1.4255	1.4176	1.1726
1.3532	1.4265	1.4666	1.4372	1.3525	1.3585	1.12
1.3866	1.444	1.451	1.4213	1.3593	1.329	1.1196
1.3779	1.368	1.3914	1.3967	1.3597	1.3767	1.103
1.3005	1.2873	1.2483	1.2673	1.2597	1.179	0.9715
1.3348	1.3025	1.3044	1.4791	1.2845	1.3006	1.0636
1.4988	1.479	1.4918	1.4084	1.3577	1.4289	1.2961
1.4863	1.515	1.5587	1.4734	1.4804	1.4673	1.3202
1.53	1.4574	1.5903	1.5675	1.5888	1.6115	1.4312
1.3678	1.3977	1.5309	1.4881	1.5462	1.4646	1.2994
1.483	1.4621	1.4925	1.4754	1.5003	1.4092	1.2208

Fig 2D Effect of different concentrations of MHY1487 on the expression of p-mTOR in Caco-2 cells.

0	1	10	100	1	10
1	0.83229946	1.27670261	1.72500593	1.83946529	2.0760021
1	1.41125814	1.74830889	1.49070927	1.44322506	1.50123141
1	1.25391384	1.18603256	1.71849208	1.75106799	1.95326602

Fig 2B p-mTOR

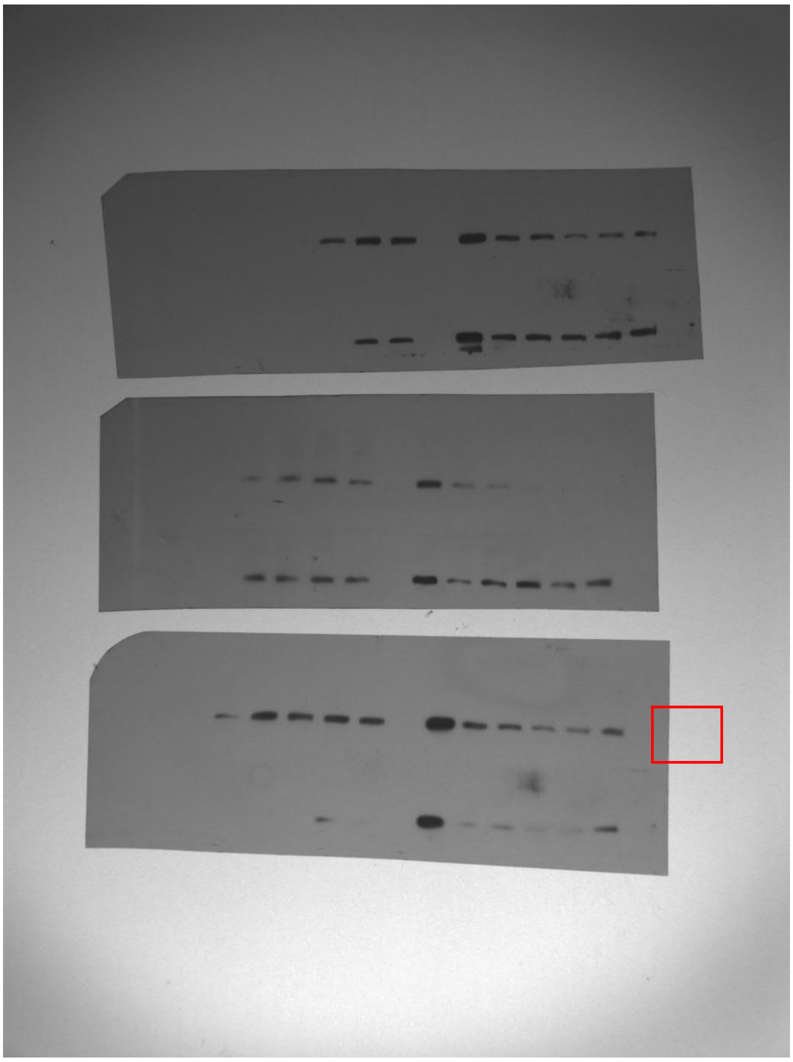

Fig 2B mTOR

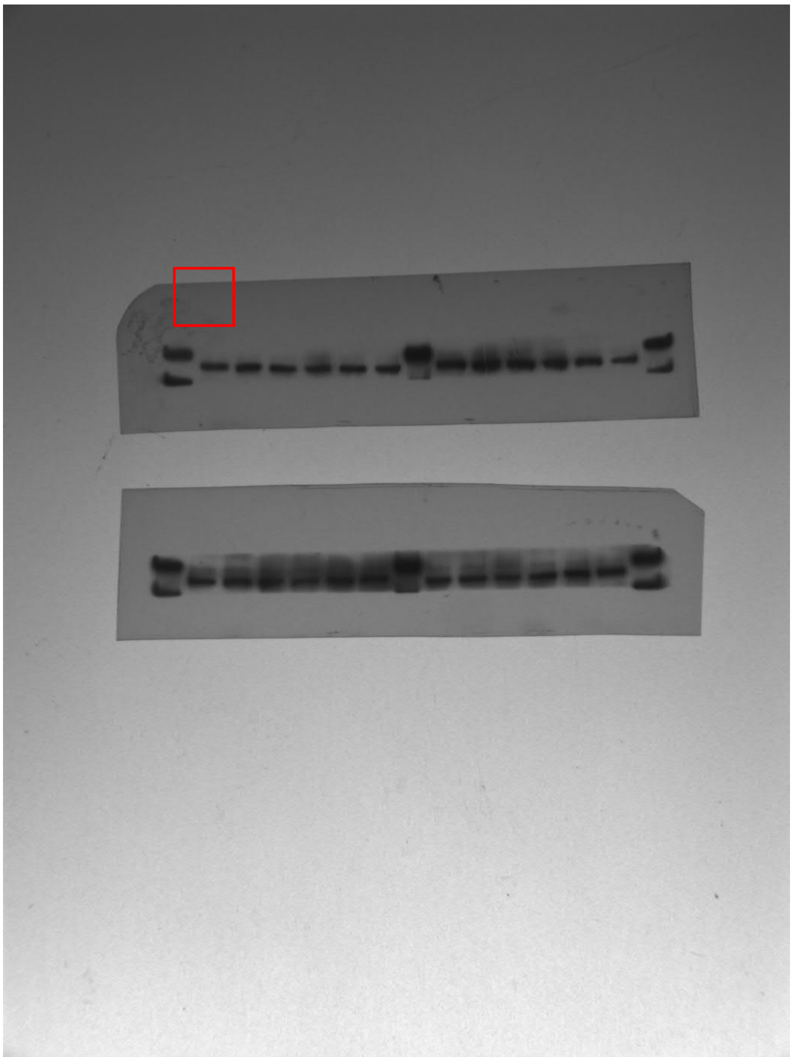

Fig 2D p-mTOR

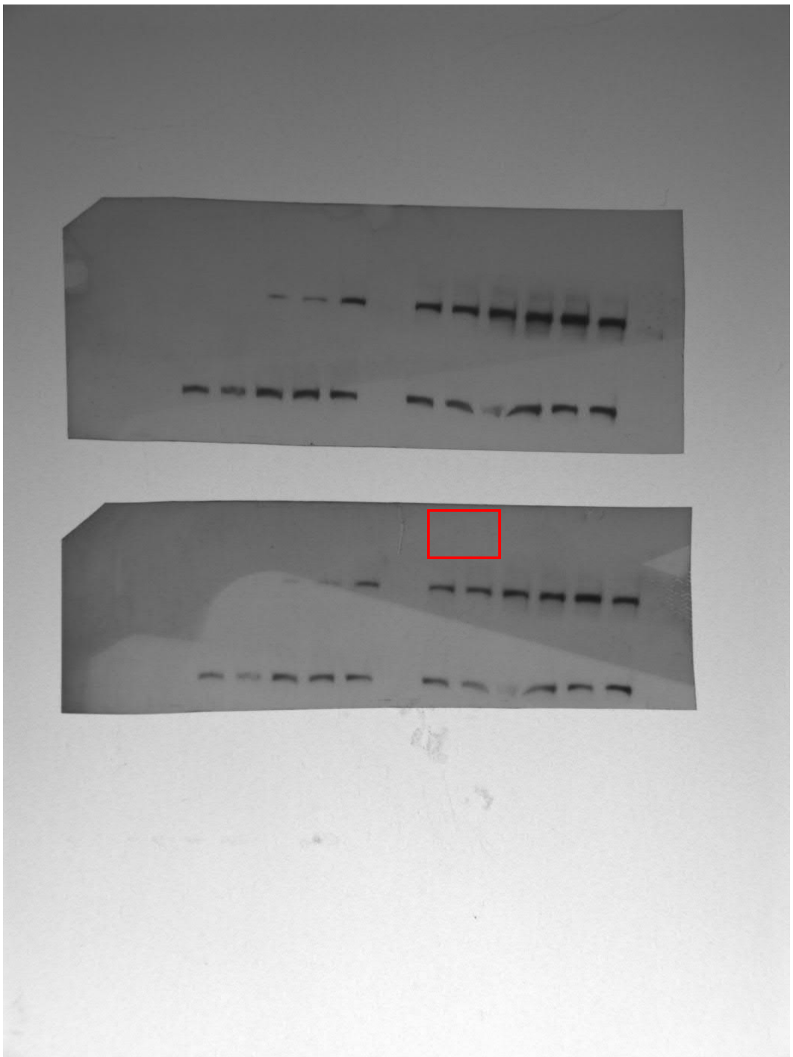

Fig 2D-mTOR

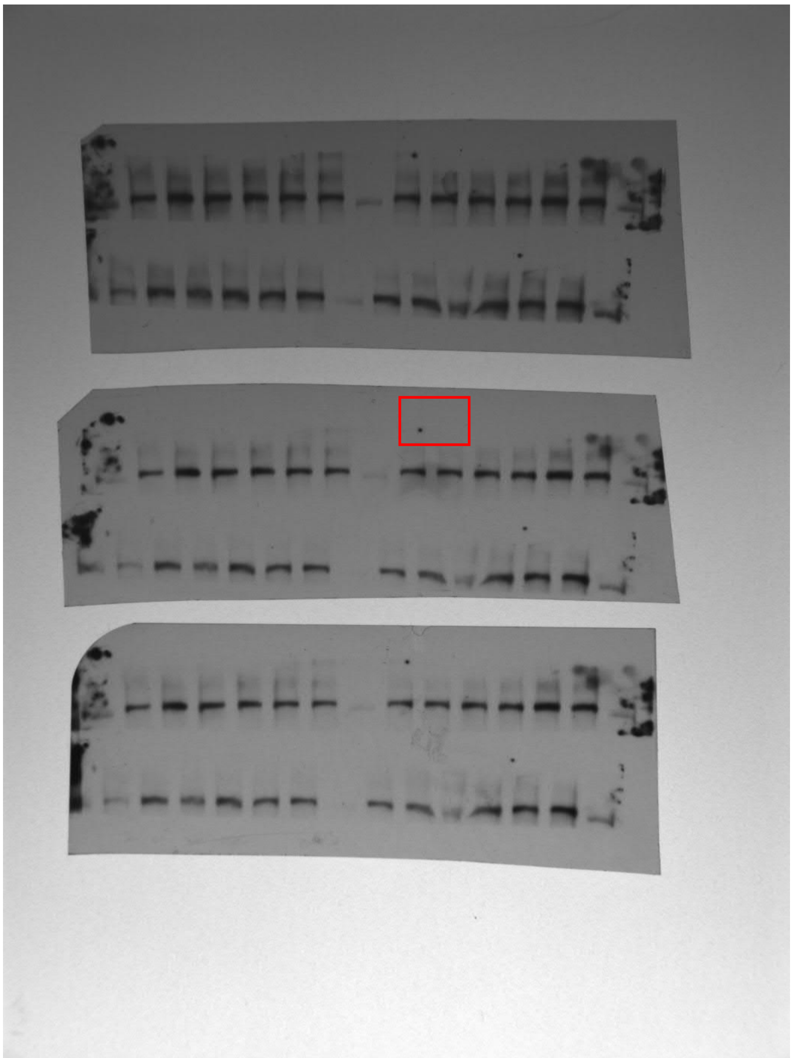

Fig3A Effects of HIF-1α activators and inhibitors on the TEER

Con	LPS	L+D	L+B
380.7	96.3	144.9	74.7
384.3	96.9	144.9	73.5
387	100.5	144	74.7
365.7	92.7	116.4	71.7
369.9	93.9	116.1	75
364.2	94.8	125.4	74.4
363.3	94.5	93.9	62.7
369.9	92.7	96	64.8
368.7	95.4	94.8	63.6

Fig 3B Effects of HIF-1α activators and inhibitors on the concentration of FD4

Con	LPS	L+D	L+B
4.2957219	125.76552	25.1354805	199.960347
3.96528176	151.539851	23.7916905	203.242719
6.93924307	129.77486	44.8076838	198.308146
1.80640613	127.307574	45.2482707	134.092611
1.71828876	153.654668	92.5452703	188.152619
7.18156585	141.031854	86.3990836	184.738071

Fig 3D the expression of HIF-1α

Con	LPS	L+D	L+B
1	1.31008283	1.63789299	0.82804374
1	1.23407658	1.54502334	1.06708269
1	1.25041881	1.73418055	0.73136275

Fig 3E the expression of ZO-1

Con	LPS	L+D	L+B
1	0.49798999	0.85780214	0.33600466
1	0.47873907	0.76077246	0.26986801
1	0.58043163	0.83720328	0.46993192

Fig 3F the expression of Occudin

Con	LPS	L+D	L+B
1	0.52387295	0.64868935	0.35507153
1	0.58628366	0.89627637	0.39758388
1	0.47581093	0.80494011	0.32914392

Fig 3G the expression of Claudin

Con	LPS	L+D	L+B
1	0.56153264	0.88562152	0.3253575
1	0.69398463	0.96451908	0.46726096
1	0.70538859	0.84881535	0.57291462

Fig 3C-HIF-1a

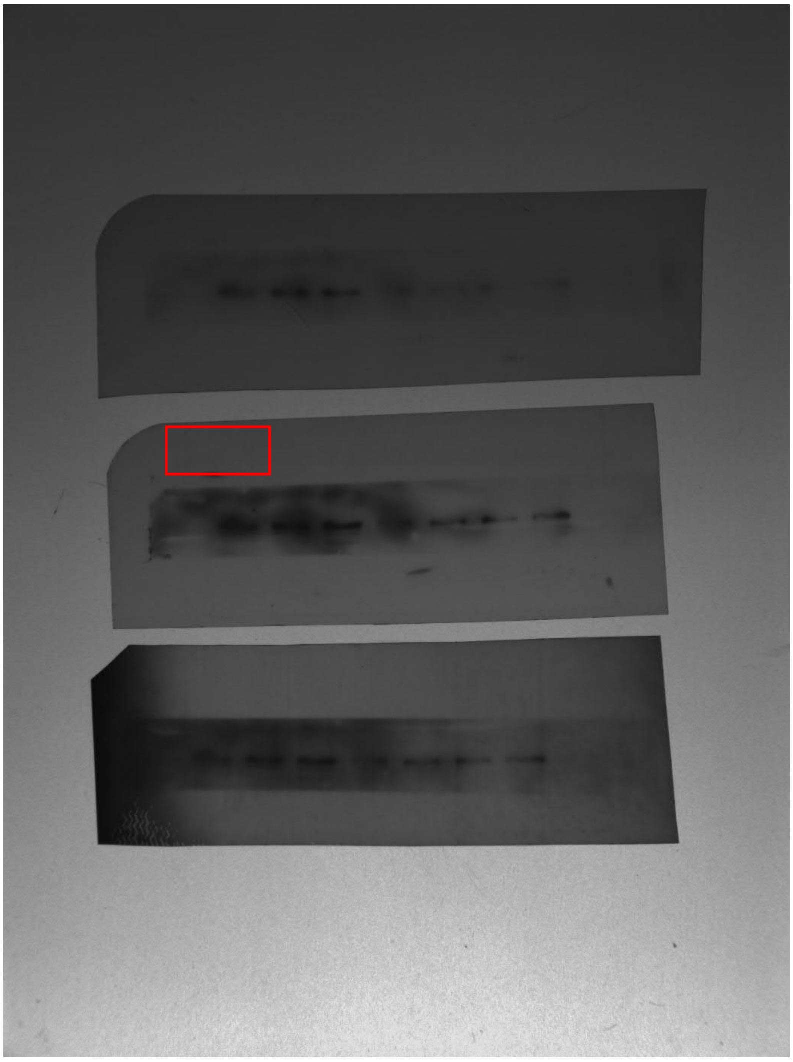

Fig 3C ZO-1

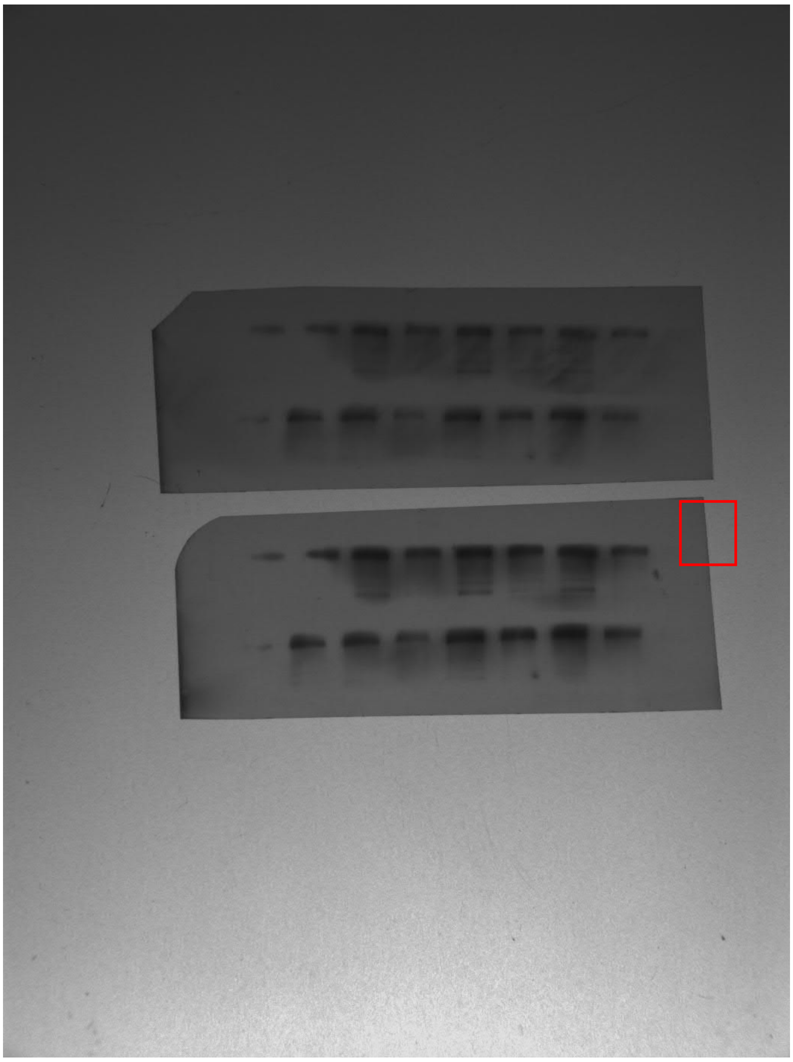

Fig 3C-Occludin

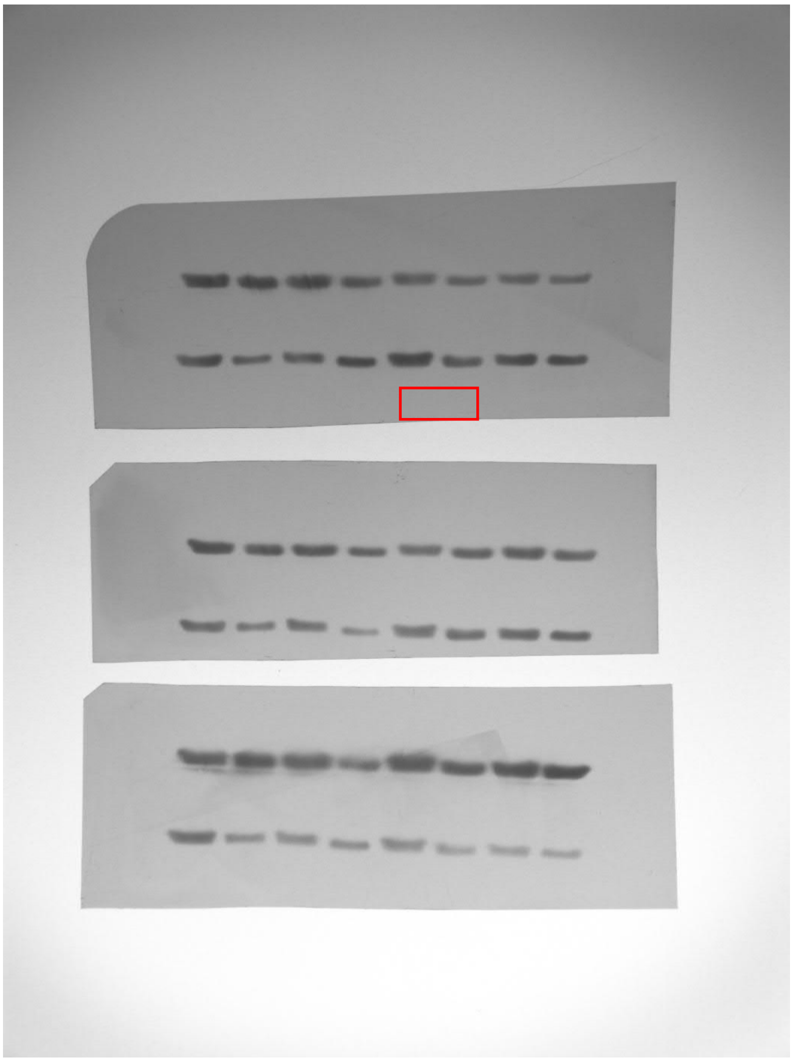

Fig 3C Caudin-1

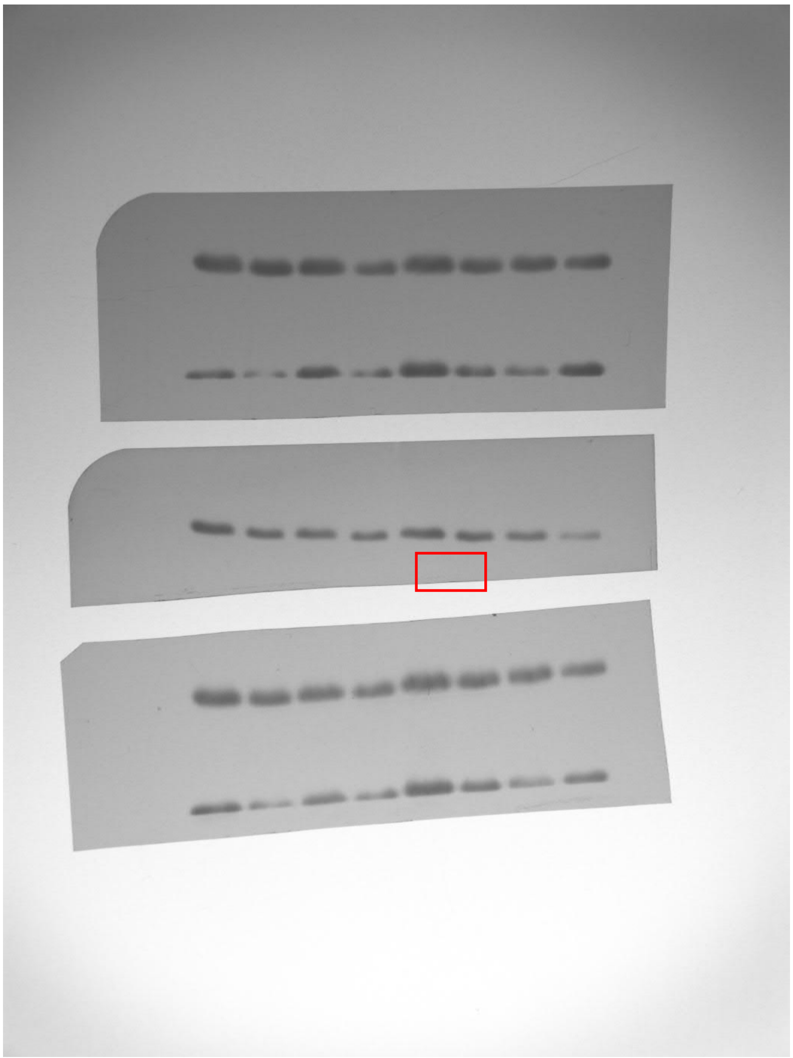

Fig 3C β -actin

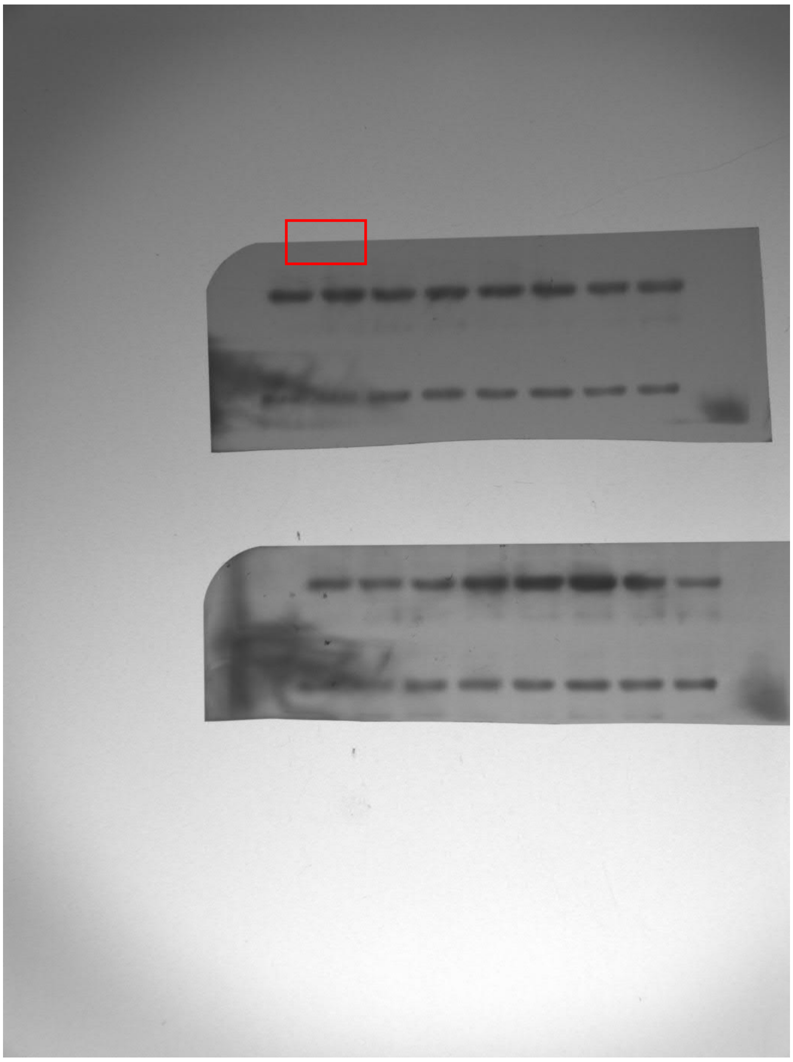

Fig4A Comparison of TEER of Caco-2 cells in each group

Con	LPS	L+R	L+M	L+R+D	L+M+B
380.7	93.3	59.7	101.4	126.9	62.7
384.3	96.9	62.4	100.8	127.2	65.4
387	94.5	60	100.5	127.5	66.9
365.7	92.7	59.4	104.1	100.8	73.5
369.9	93.9	57.3	103.8	103.5	75.6
364.2	94.8	55.2	101.7	100.2	79.5
363.3	94.5	64.2	106.8	115.5	93.9
369.9	92.7	62.4	110.4	117.6	93.6
368.7	95.4	63.3	110.1	114.9	93.9

Fig 4B FD4

Con	LPS	L+R	L+M	L+R+D	L+M+B
4.2957219	125.76552	188.284795	79.7902807	101.1807728	205.3575362
3.96528176	151.539851	190.619906	95.7174957	95.93778914	191.1045513
6.93924307	129.77486	188.262766	121.557915	53.26695158	205.9302992
1.80640613	127.307574	150.768824	113.891704	100.6961272	232.3875402
1.71828876	153.654668	145.239459	102.788915	92.41309424	208.1332335
7.18156585	141.031854	200.577169	98.0966648	109.0672776	141.7367934

Fig 4D

Con	LPS	L+R	L+M	L+R+D	L+M+B
1	1.33597702	0.48034364	1.4826197	0.73334576	1.56932694
1	1.22918457	0.46472539	1.4856458	0.64179476	1.50354667
1	1.31677943	0.59248019	1.6145538	0.95707299	1.72194775

Fig 4E

CON	LPS	L+R	L+M	L+R+D	L+M+B
1	1.15753212	0.33713426	1.20934191	0.21659425	1.36323102
1	1.15406877	0.07739545	1.37532113	0.30342944	1.42943544
1	1.09259185	0.34626322	1.26349311	0.34814281	1.55273112

Fig 4F

CON	LPS	L+R	L+M	L+R+D	L+R+B
1	1.87549951	0.06518935	2.12675217	3.67254736	0.09905473
1	1.60018635	0.5930111	2.98135662	4.1527301	0.53886431
1	1.90665267	0.62678468	2.20898849	2.87725781	0.80726034

Fig 4G

Con	LPS	L+R	L+M	L+R+D	L+M+B
1	0.64017417	0.49549112	0.66207574	0.74119327	0.38880925
1	0.5831851	0.36236149	0.65640973	0.70012242	0.27577277
1	0.53732561	0.37799099	0.71162709	0.77665191	0.30225515

Fig 4H

Con	LPS	L+R	L+M	L+D+R	L+B+M
1	0.71873309	0.5781235	0.81200199	0.87936218	0.44233902
1	0.68666068	0.29852492	0.78787845	0.97036314	0.33379455
1	0.69136142	0.61338184	0.77265333	0.86071257	0.38062781

Fig 4I

Con	LPS	L+R	L+M	L+R+D	L+M+B
1	0.700927846	0.333124362	0.71774691	0.917442473	0.413470709
1	0.700251202	0.574048222	0.73516525	0.871478747	0.490058714
1	0.678126329	0.549782469	0.94263989	0.904663836	0.538857198

Fig 4C-p-mTOR

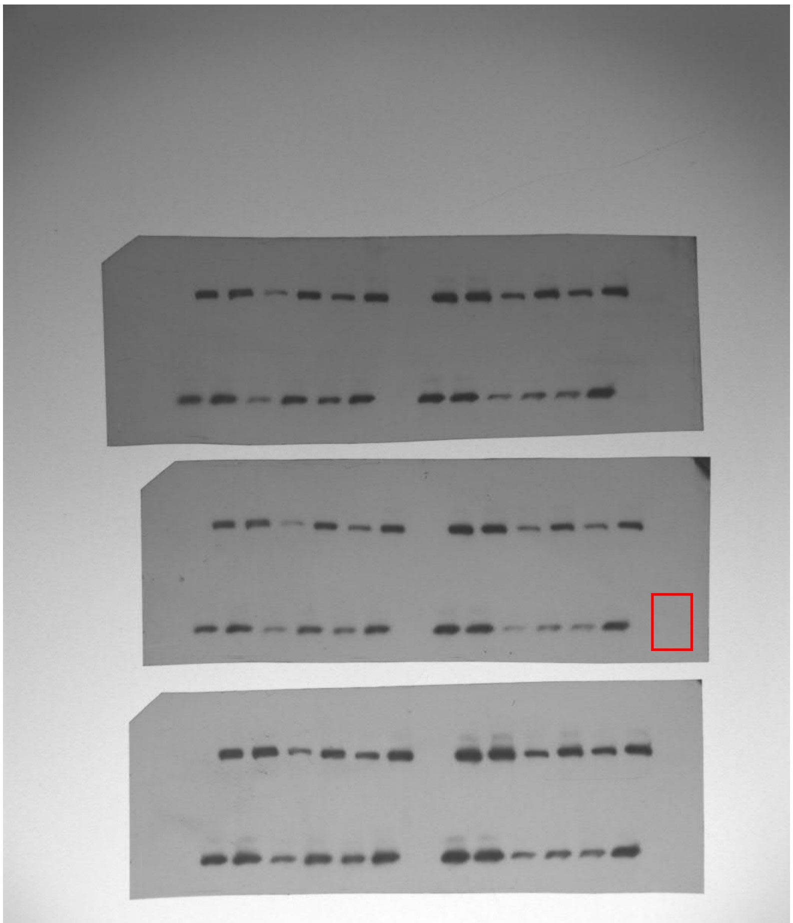

Fig 4C-mTOR

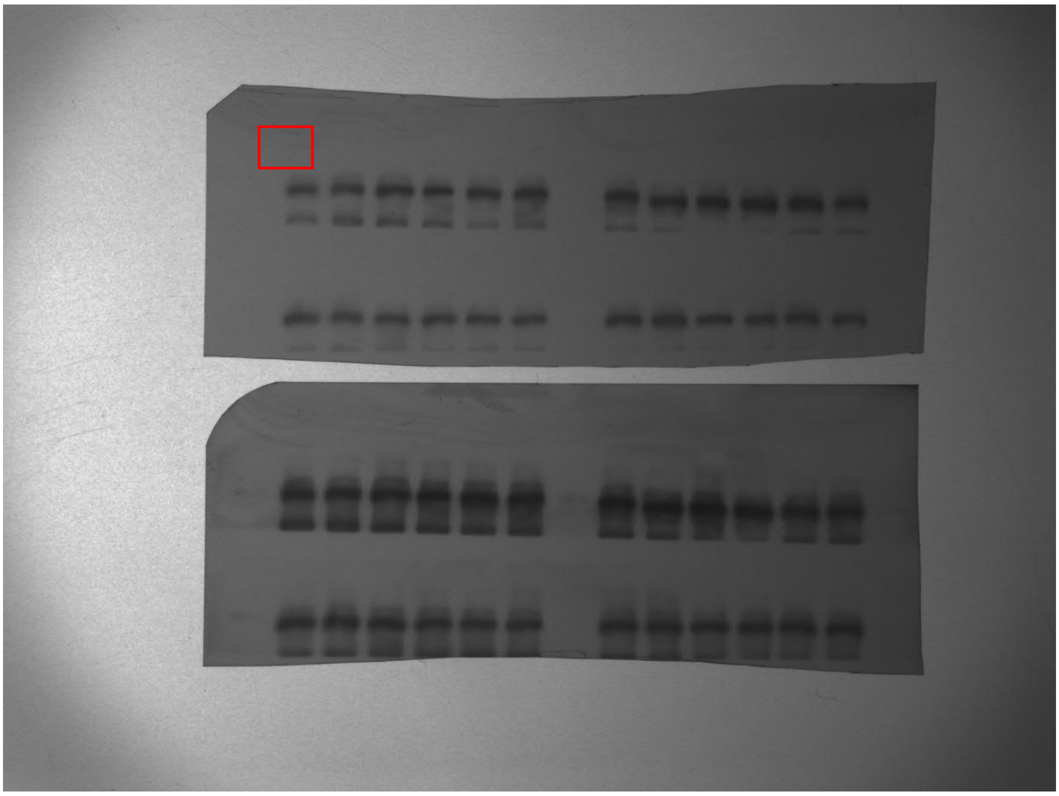

Fig 4C-p-P70S6K

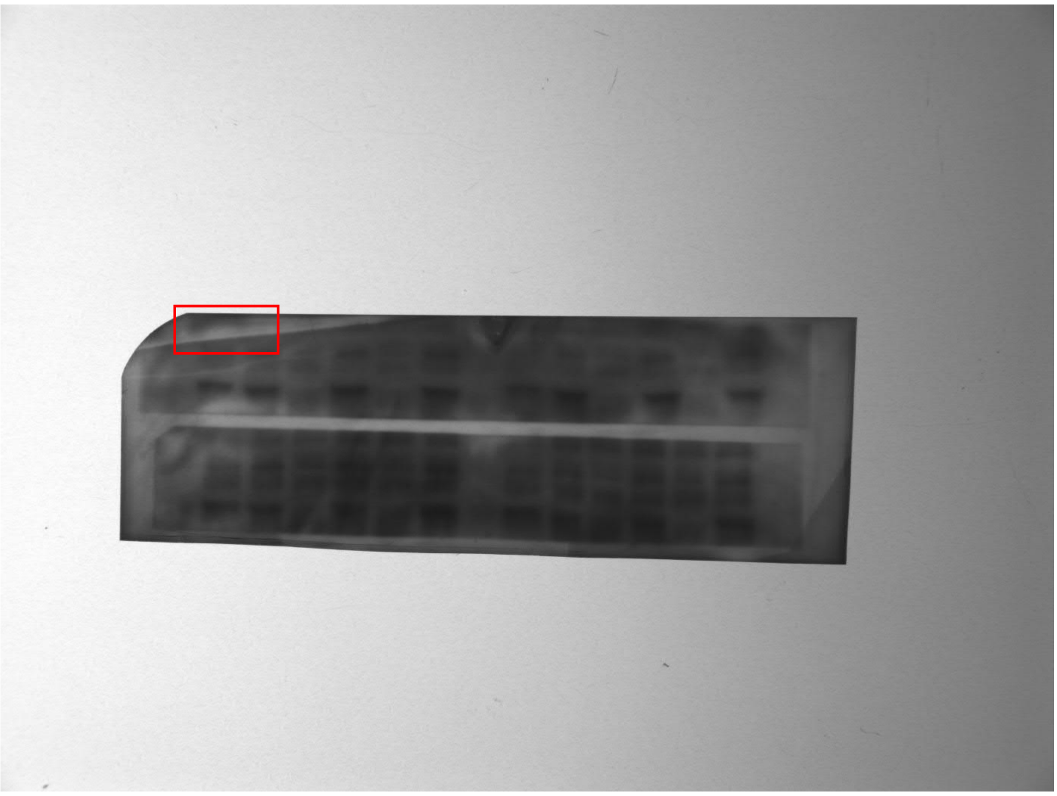

Fig 4C P70S6K

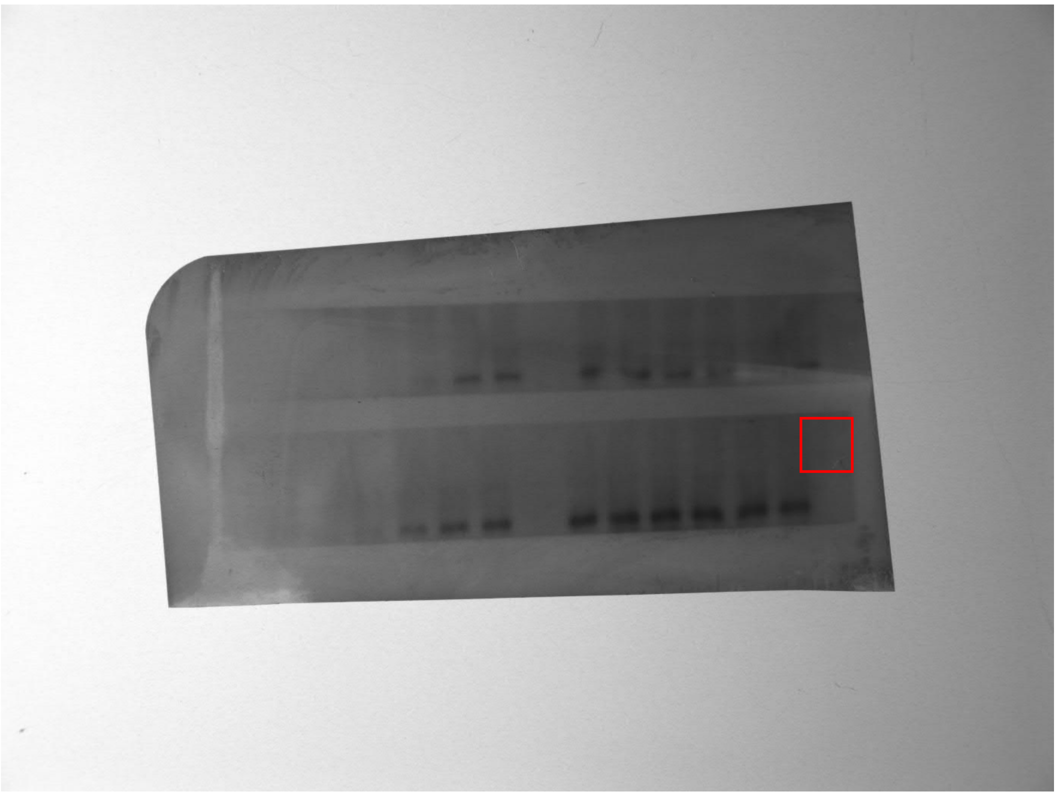

Fig 4C HIF-1a

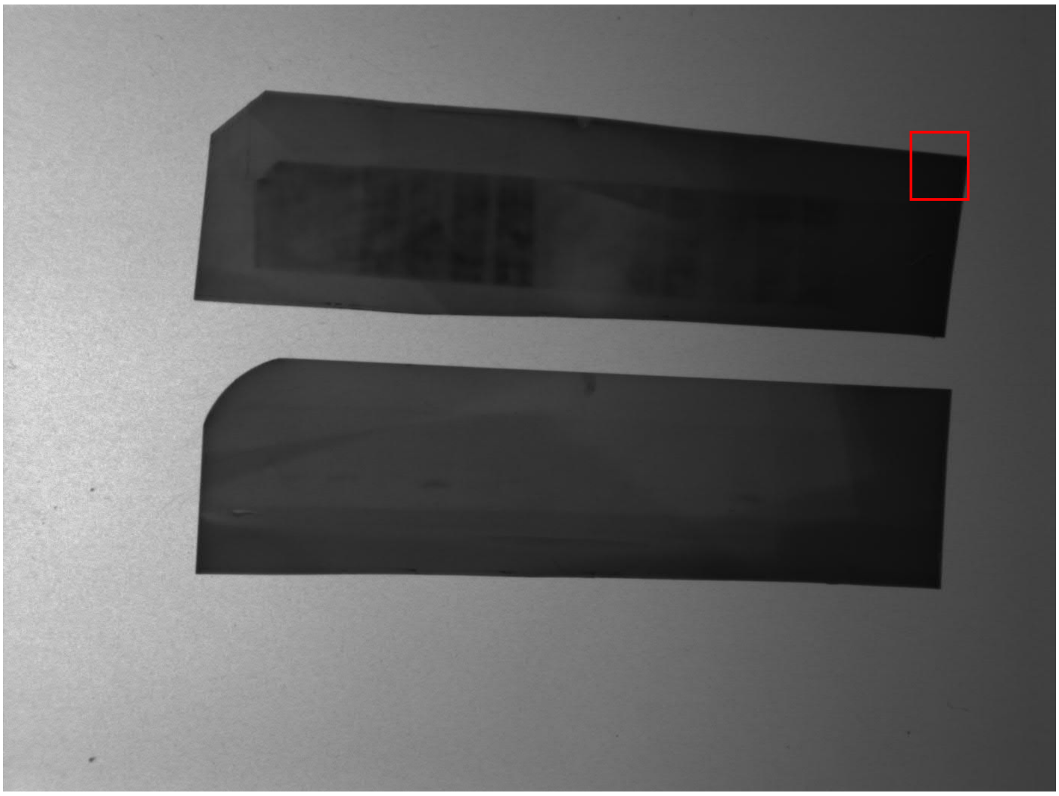

Fig 4C ZO-1

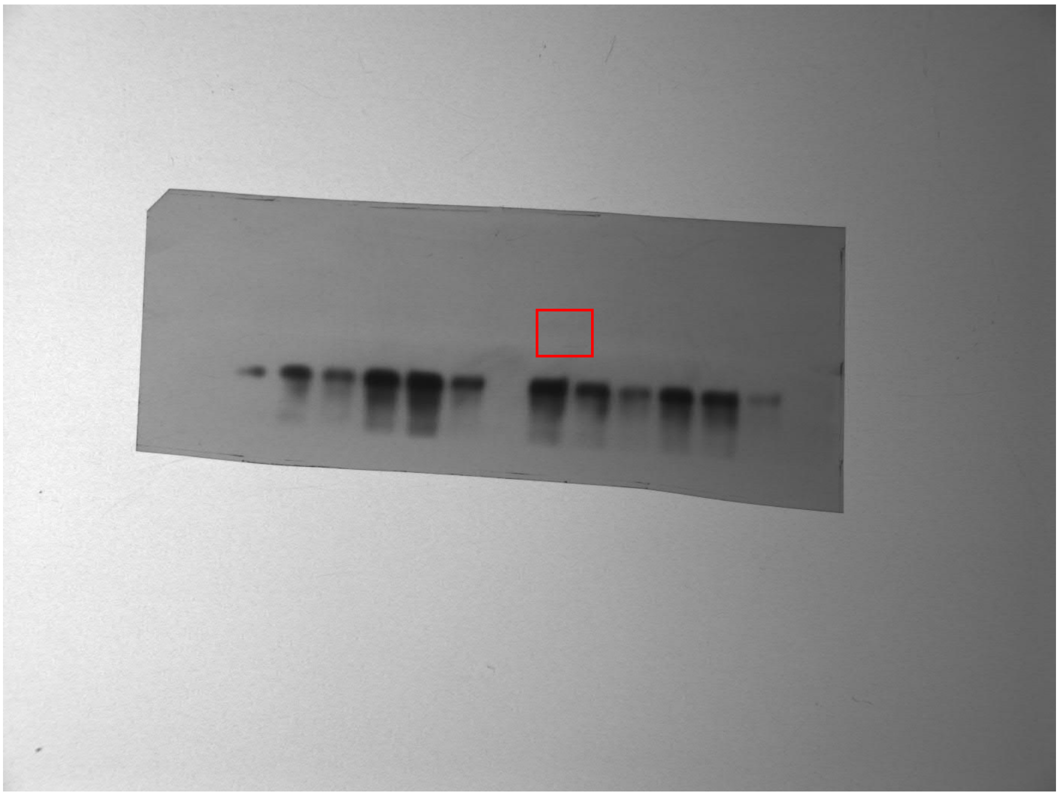

Fig 4C Occludin

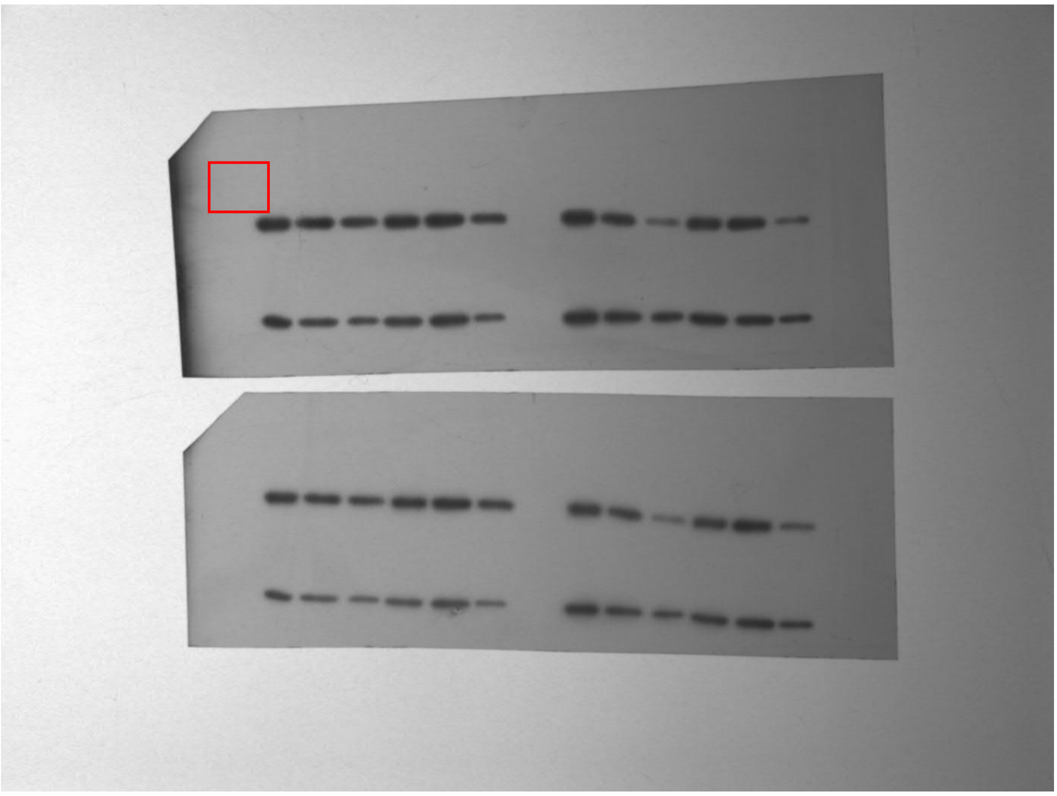

Fig 4C Claudin-1

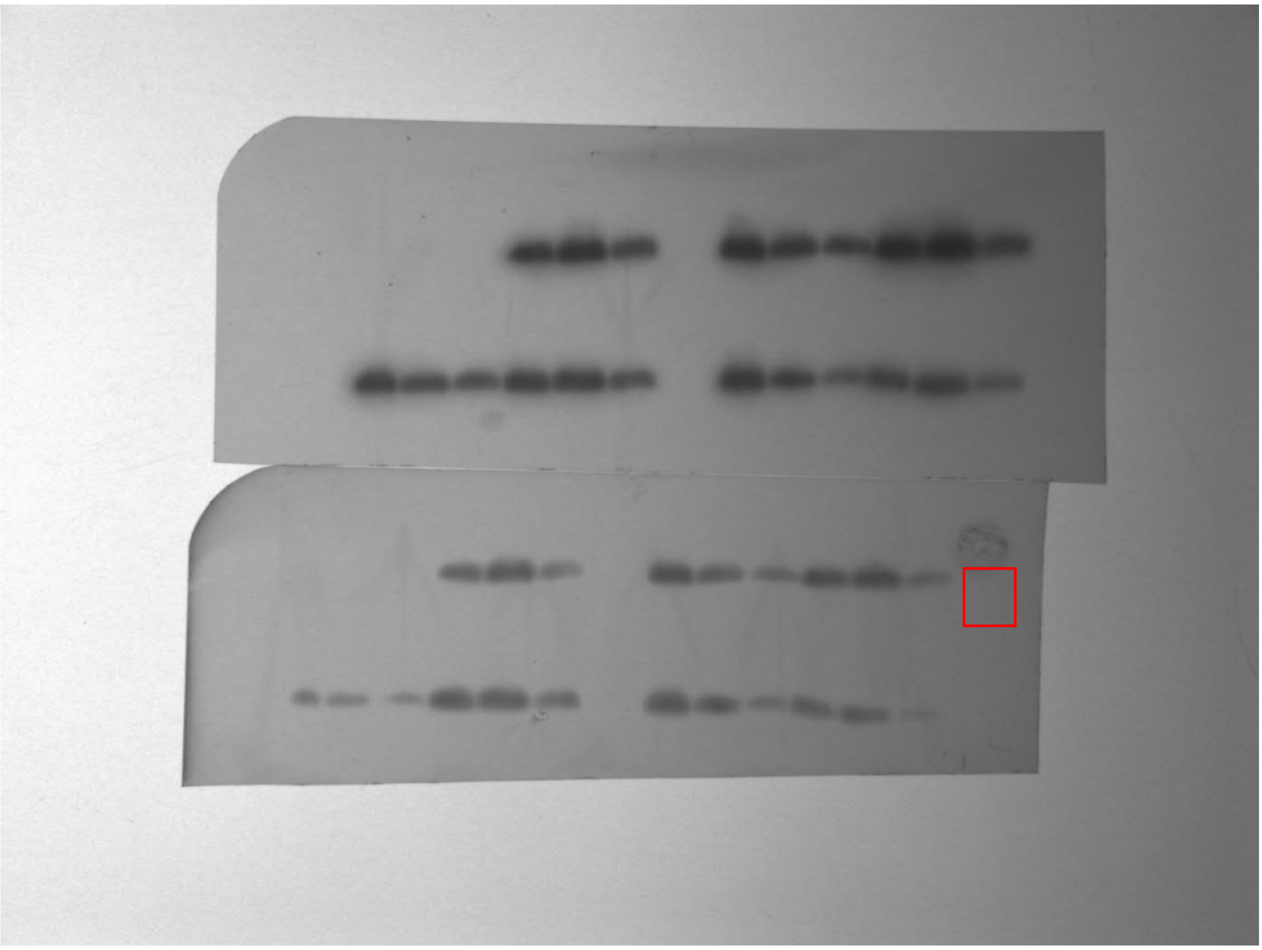

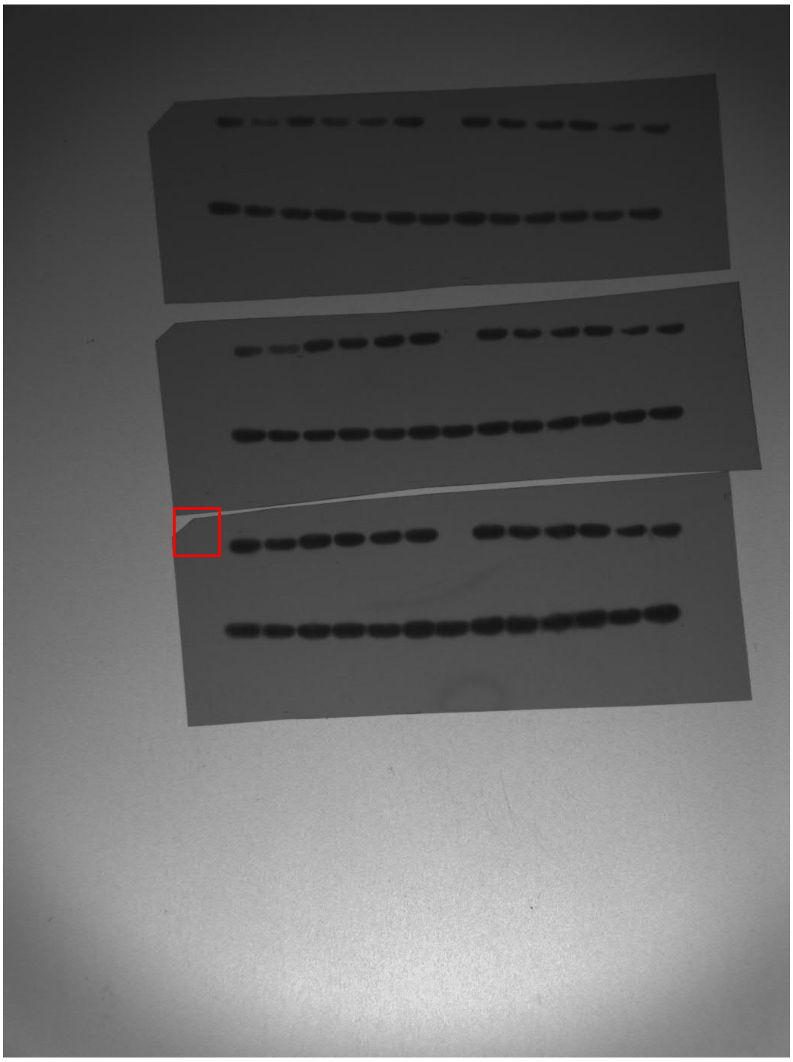

Fig 4C β -actin
